# Auxin affects gene editing efficiency through regulating chromatin accessibility and plant regeneration process

**DOI:** 10.1093/hr/uhaf240

**Published:** 2025-09-03

**Authors:** Zhaoyuan Lian, Tao Jiang, Yufei Liang, Wanxing Hu, Huimin Peng, Hanghang Zhang, Haijun Gong, Chunxiang You, Guiluan Wang, Li Liu, Heqiang Huo

**Affiliations:** College of Horticulture, Northwest A&F University, Yangling, Shaanxi 712100, China; Department of Horticultural Sciences, Mid-Florida Research and Education Center, IFAS, University of Florida, Apopka, FL 32703, USA; Department of Horticultural Sciences, Mid-Florida Research and Education Center, IFAS, University of Florida, Apopka, FL 32703, USA; College of Horticulture, Northwest A&F University, Yangling, Shaanxi 712100, China; College of Horticulture, Northwest A&F University, Yangling, Shaanxi 712100, China; College of Horticulture, Northwest A&F University, Yangling, Shaanxi 712100, China; College of Horticulture, Northwest A&F University, Yangling, Shaanxi 712100, China; College of Horticulture, Northwest A&F University, Yangling, Shaanxi 712100, China; College of Horticulture Science and Engineering, Shandong Agriculture University, Tai-An 271018, Shandong, China; Department of Horticultural Sciences, Mid-Florida Research and Education Center, IFAS, University of Florida, Apopka, FL 32703, USA; State Key Laboratory of Wheat Improvement, Peking University Institute of Advanced Agricultural Sciences, Shandong Laboratory of Advanced Agricultural Sciences in Weifang, Shandong 261325, China; State Key Laboratory of Biocatalysis and Enzyme Engineering, Hubei Collaborative Innovation Center for Green Transformation of Bio-Resources, Hubei Key Laboratory of Industrial Biotechnology, School of Life Sciences, Hubei University, Wuhan, Hubei, China; Department of Horticultural Sciences, Mid-Florida Research and Education Center, IFAS, University of Florida, Apopka, FL 32703, USA

## Abstract

Improving gene editing efficiency has been a prominent research focus with the increasing application of CRISPR/Cas9 in crop genetic enhancement. In this study, we demonstrated that increasing exogenous auxin levels during *in vitro* tissue culture significantly enhances gene editing efficiency, leading to a higher frequency of functionally edited T_0_ plants. While higher auxin levels promoted callus growth, it also delayed shoot initiation and slightly decreased shoot regeneration. Subsequent RNA-Seq analysis revealed significant alterations in the expression of plant developmental regulatory genes and chromatin remodeling genes at two plant regeneration stages. Further analysis using nuclei staining and Transposase-Accessible Chromatin using sequencing showed that excessive auxin resulted in a more relaxed chromatin structure in callus cells, thus enhancing the genomic DNA accessibility to Cas9. Additionally, the prolonged growth period of dedifferentiated callus cells and the delay in shoot initiation likely provided additional time for Cas9 to exert its function, explaining the improved gene editing efficiency due to excessive auxin application. To mitigate the inhibitory effects of excessive auxin on shoot regeneration, a ‘two-phase’ culture strategy was developed and validated using tomatoes, in which the explants were first cultured in media containing excessive auxin to promote calli growth and gene editing, then transferred to the media with lower auxin concentrations to promote the following shoots regeneration. Overall, our research has revealed novel aspects of auxin function in gene editing, offering new insights and a theoretical basis for future studies. Furthermore, the proposed culture method could accelerate the application of gene editing across various plant species.

## Teaser

**One-sentence summary:** A modest increase in auxin concentration during *in vitro* tissue culture enhances the generation of functionally edited plants in the T₀ generation by increasing chromatin accessibility and slowing process of plant regeneration.

## Introduction

The advent of the CRISPR/Cas9 genome editing system and its variants has revolutionized crop improvement in agriculture [1, 2]. This cutting-edge technology enables precise and diverse genetic modifications, including targeted editing, base substitutions, small insertions/deletions, and targeted gene regulation, through RNA-guided tools, thereby facilitating the development of desired traits and novel trait combinations, often without the need for foreign gene introduction [3]. Consequently, CRISPR genome editing typically generates non-GMO products with targeted mutations in annual seeded crops because the transgene can be easily eliminated through seed segregation allowing desirable mutations to be maintained. This approach simplifies regulatory scrutiny, increases the likelihood of approval success, and enhances consumer acceptance of gene-edited crop products [4–6]. Despite these significant advancements, the efficacy of gene editing varies considerably across plant species. Some species, such as Chrysanthemum (*Chrysanthemum morifolium*), frequently exhibit chimeric editing events, leading to unstable inheritance and low gene editing efficiency [7, 8]. Additionally, a considerable number of gene-edited T_0_ plants are heterozygous, necessitating extensive effort to identify nontransgenic homozygous progeny. This challenge is particularly pronounced in plant species that are vegetatively propagated and polyploid with complex genetic backgrounds, where sexual seeds may not be produced or desirable traits may be lost during seed segregation [9, 10].

Since CRISPR/Cas9 system was initially applied to Arabidopsis (*Arabidopsis thaliana*) and tobacco (*Nicotiana tabacum* L*.*) in 2013 [11], enhancing gene editing efficiency in plants has been a focal area of research. Various strategies have been developed to increase gene editing efficiency, which can be categorized as follows: (i) expanding PAM recognition sequences through the development and modification of Cas9 variants [12]; (ii) optimizing Cas9 expression using different promoters or using intron-containing Cas variants. For example, replacing the CaMV35S promoter with an Arabidopsis UBQ10 promoter quadrupled gene editing efficiency in *Arabidopsis* and *Medicago truncatula* [13]; Additionally, the inclusion of multiple introns into the Cas9 or Cas12 coding sequence was reported to dramatically improve the editing efficiency in *A. thaliana* [14, 15]; (iii) employing multiple gRNAs to simultaneously edit several homologous genes. For instance, improved salt tolerance in soybean (*Glycine max*) was improved by targeting six *GmAITR* homologous genes [16]; (iv) delivering Cas9 and gRNAs into plant reproductive organs efficiently using nanoparticles or virus-mediated systems to produce gene-edited seeds [17, 18]; (v) overexpressing developmental regulatory genes to enhance *in vitro* plant regeneration during tissue culture, beneficial for species with poor regeneration capabilities [19]; (vi) injecting wounded plants with *A. tumefaciens* that carry developmental regulatory genes (e.g. *WUS2/IPT* or *WUS2/STM*) to induce direct shoot regeneration from the wounded sites as demonstrated by Maher et al. [20], where *pds* mutant shoots were successfully induced from soil-grown tobacco. In summary, the first three strategies directly improve gene editing efficiency through engineering CRISPR/Cas9 system, while the last three indirectly enhance gene editing efficiency by accelerating mutant acquisition.

Eukaryotic DNA is packaged into complex chromatin structures within the nucleus, with DNA tightly wound around histone proteins. During DNA transcription and replication, chromatin undergoes dynamic structural remodeling, transiently relaxing its compact configuration to enable interactions with transcription factors, RNA polymerases, and other regulatory proteins. This transition of chromatin from a closed to an open state, also known as chromatin accessibility, is essential for gene regulation [21, 22]. Interestingly, chromatin accessibility has been reported to be closely associated with gene editing efficiency. For example, heterochromatin, characterized by its condensed spatial structure, restricts chromatin accessibility. Kim, et al. [23] demonstrated that constitutive heterochromatin reduces CRISPR-Cas9 editing efficiency in the HEK293T cell line. Similarly, Chari, et al. [24] found that insertions and deletions (indels) generated by CRISPR/Cas9 editing occur more frequently in the open chromatin regions of the human genome. Similar findings have been reported in rice (*Oryza sativa* L.) as well [25]. To address these challenges, strategies have been developed to artificially increase chromatin accessibility at target sites to improve gene editing efficiency. For instance, Liu et al. [25] engineered a fusion protein, Cas9-TV, by fusing Cas9 with a synthetic transcriptional activator. This construct enhanced chromatin accessibility at target loci, resulting in a higher gene editing efficiency. Moreover, the inclusion of a ‘dead sgRNA’ (dsgRNA) cassette, which binds to genomic DNA without causing double-strand break, further enhanced chromatin accessibility near target sites and the editing efficiency of Cas9-TV.

Histone acetylation, which neutralizes the positive charge of the histone tails and weakens their affinity for DNA, is another well-documented mechanism for promoting chromatin openness [26, 27]. Leveraging this principle, researchers have applied different histone deacetylase inhibitors (HDACis) to cultured cells to increase chromatin accessibility, thereby resulting in varying degrees of improvement in CRISPR/Cas9 editing efficiency [28–30]. For example, Wang et al. [31] demonstrated that adding 2.5 mM nicotinamide to culture media increased gene editing efficiency in hexaploidy wheat to 30.3% in immature embryos and 13.3% in mature embryos compared to 0% efficiency observed in the control group without nicotinamide. Despite the significant potential for increasing chromatin accessibility to improve the gene editing efficiency, most studies have focused on achieving this through complex biotechnological designs.

Recent findings demonstrate that auxin signaling modulates chromatin accessibility through epigenetic mechanisms involving histone acetylation, histone methylation, and nucleosome remodeling [32, 33]. Auxin influences the activity of histone acetyltransferases (HATs) such as *GCN5* and histone deacetylases (HDACs) including *HDA6*, thereby regulating transcriptional competence at target loci through dynamic histone acetylation states [34–36]. Additionally, auxin-responsive transcription factors, particularly AUX/IAA and ARF proteins, interact with co-repressors like *TOPLESS*, which recruit HDACs to establish a repressive chromatin environment [37, 38]. Beyond acetylation, auxin also impacts histone methylation through crosstalk with *Polycomb Repressive Complex 2 (PRC2)*, leading to dynamic regulation of H3K27me3 during developmental transitions [39–41]. Moreover, auxin has been shown to promote chromatin remodeling at key developmental loci, including *PLETHORA* genes, through histone acetylation-dependent mechanisms. This remodeling facilitates the transcriptional activation of downstream auxin biosynthetic genes such as *YUCCA1*, establishing a positive feedback loop that integrates epigenetic regulation with hormonal signaling [40, 42]. Such hormone-driven epigenetic changes contribute to increased chromatin accessibility at gene loci, including those targeted in CRISPR/Cas9 genome editing, potentially enhancing editing efficiency by modulating the chromatin environment.

In our study, we discovered that modestly increasing the concentration of auxin during *in vitro* tissue culture influences chromatin accessibility and significantly improves the generation of functionally edited plants in the T₀ generation. Furthermore, we observed that plant regeneration progress was altered by the increased auxin concentration in the culture media. Enhanced callus growth and delayed shoot initiation may offer a longer time window for the Cas9 activity during the rapid division of dedifferentiated cells, thereby indirectly improving gene editing efficiency. Consequently, a significantly greater number of functionally edited plants in the T₀ generation, which was a finding with profound significance for accelerating gene editing breeding programs. This is particularly valuable for plant species that reproduce asexually, possess complex genetic backgrounds, or exhibit polyploidy.

Our conclusions were corroborated by histological examination, RNA-seq and Assay for Transposase-Accessible Chromatin using sequencing (ATAC-seq) analyses of explants cultured in media containing different concentrations of auxin-naphthaleneacetic acid (NAA). These findings not only open a new avenue for enhancing gene editing efficiency in plants by modulating chromatin accessibility but also highlight the potential of leveraging plant hormones and developmental processes to optimize gene editing methods and advance the application of CRISPR technology in crop improvement programs.

## Results

### Effects of NAA concentrations on plant regeneration in tobacco

**Figure 1:**
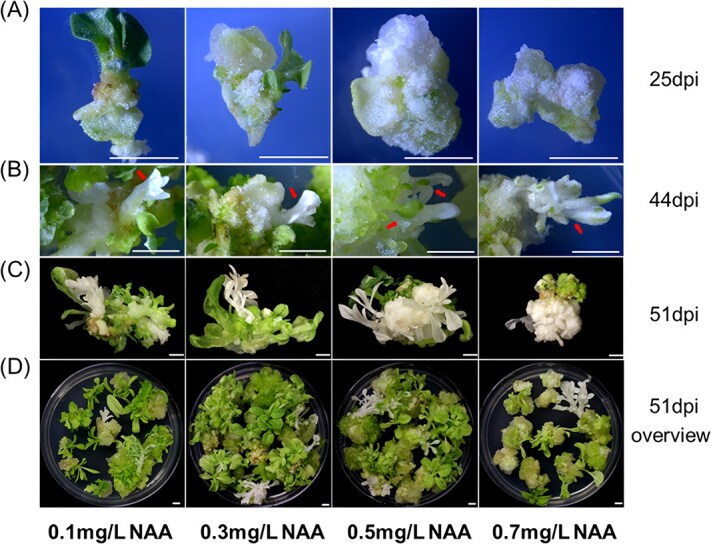
Effects of NAA concentrations on the tobacco regeneration. (A) Morphology of explants at 25 days postinoculation (dpi). Shoot regeneration was observed in the 0.1 mg/L and 0.3 mg/L NAA groups, but not in the 0.5 mg/L and 0.7 mg/L NAA groups. (B) Albino shoots resulting from gene editing of *NbPDS3* at 44 dpi, indicated by the arrows. (C and D) Fully developed shoots observed at 51 dpi, with more pronounced callus formation in the 0.5 and 0.7 mg/L NAA groups. Scale bar = 1 cm.

Sterilized tobacco seeds were grown on preculture medium (MS without hormones and antibiotics). After two weeks, fully extended cotyledons were excised and used for the subsequent experiment. We then investigated the impact of a range of NAA concentrations (0.1, 0.3, 0.5, and 0.7 mg/L) in combination with 2 mg/L 6-benzyladenine (6-BA) on tobacco regeneration following infection with *A. tumefaciens* harboring the plasmid for editing phytoene desaturase 3 (*PDS3*) (Supplementary Fig. S1). Our observations revealed that shoot regeneration occurred significantly earlier on callus and shoot induction media (MS + 2 mg/L 6-BA +100 mg/L kanamycin +100 mg/L Timentin + varying concentrations of NAA) supplemented with lower NAA concentrations. Specifically, shoot regeneration was already visibly detectable at 25 days postinfection (dpi) in the 0.1 and 0.3 mg/L NAA groups, but no visible albino mutants were observed. By contrast, large masses of calli formed in the 0.5 and 0.7 mg/L NAA groups, with no visible shoots (Fig. 1A). By 44 dpi, albino shoot initiation was observed across all treatment groups, as indicated by the red arrows (Fig. 1B). By 51 dpi, most regenerated shoots were fully developed, and gene-edited shoots with albino phenotypes were readily noticeable in all groups. However, callus masses were significantly larger in the 0.5 and 0.7 mg/L NAA groups compared to the 0.1 and 0.3 mg/L NAA groups (Fig. 1C and D).

### Effects of NAA concentrations on gene editing efficiency in tobacco

To examine and visualize gene editing events, we selected *PDS3* gene as our editing target in tobacco, and two gRNAs were selected to target *NbPDS3* as shown in Supplementary Fig. S1. The expression of *PDS* is essential for chloroplast development [43], and its disruption typically causes varying degrees of albinism in the regenerated shoots during *in vitro* tissue culture. This albino phenotype facilitates the investigation of how different NAA concentrations affect gene editing efficiency.

**Figure 2:**
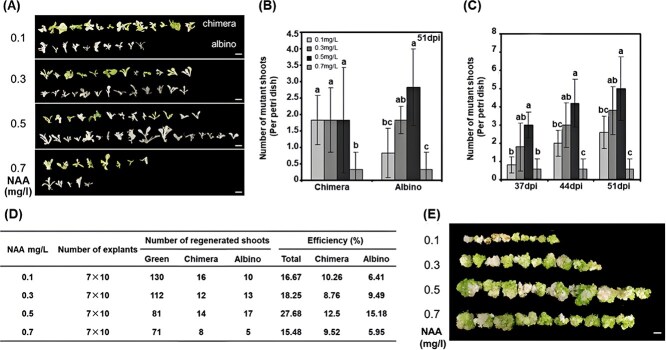
Effects of NAA concentrations on gene editing efficiency in tobacco. (A) Chimeric (top) or albino shoots (bottom) from explants treated with different NAA concentrations. (B) Quantitative comparison of mutant shoots per petri dish across different NAA concentrations, showing chimera (left) and complete albino shoots (right). (C) Quantitative comparison of total mutant shoots per petri dish from different NAA concentrations at 37-, 44- and 51-days postinoculation (dpi). (D) Total number of regenerated shoots and mutant shoots from different NAA concentrations, along with corresponding efficiencies. Seven replicates petri dish were applied to each NAA concentration (0.1, 0.3, 0.5, and 0.7 mg/L), with each replicate consisting of 10 explants (cotyledons). (E) The remaining calli from the same explants in (A). Error bars in B and C represent the standard error values, with significant difference denoted by letters among different groups (ANOVA-LSD, *P* <0.05), Scale bar = 1 cm.

Our results suggested that increasing exogenous auxin during *in vitro* regeneration significantly enhanced the frequency of shoots displaying the albino phenotype, consistent with disruption of all functional alleles at the targeted locus. In brief, the 0.5 mg/L NAA treatment produced the highest number of albino mutants (regenerated shoots with complete albino phenotypes), followed by the 0.3 and 0.1 mg/L NAA groups. No significant differences were observed in the number of chimeric mutants among 0.1, 0.3, and 0.5 mg/L NAA groups (Fig. 2A–D). However, the 0.7 mg/L NAA showed severely inhibited shoot regeneration, resulting in the lowest production of both mutant types (Fig. 2A–D). Mutant numbers per petri dish were measured at 37, 44, and 51 days postinfection (dpi) revealed consistent trends over time, with the 0.5 mg/L NAA group consistently producing the most mutants. However, this difference was not statistically significant relative to the 0.3 mg/L group (Fig. 2C). To further illustrate the gene editing outcomes, albino or chimeric shoots from 10 explants per treatment were excised and displayed (Fig. 2A), and the remaining callus tissues from the same explants were also arranged for visualization in Fig. 2E. Interestingly, callus size significantly increased with higher NAA concentrations, except in the 0.7 mg/L NAA group, where it was slightly smaller than in the 0.5 mg/L NAA group (Fig. 2E and Supplementary Fig. S2).

Editing efficiency was calculated based on the total number of regenerated shoots and visible mutants (the regenerated shoots with visible mutant phenotypes including complete albino and chimera) recorded at 51dpi (Fig. 2D). Despite a reduction in the number of regenerated shoots, the 0.5 mg/L NAA group achieved the highest visible mutant efficiency (27.68%), comprising 12.50% chimeric mutants and 15.18% albino mutants (Fig. 2D). Notably, the albino mutant efficiency (15.18%) was significantly higher than other treatment groups, being 2.6 times greater than the 0.7 mg/L NAA group (5.95%), 2.4 times greater than the 0.1 mg/L NAA group (6.41%), and 1.6 times greater than the 0.3 mg/L NAA group (9.49%). PCR sequencing confirmed putative gene editing events by detecting insertions or deletions in selected sequencing samples, including a total of 25 samples each from the 0.1 and 0.5 mg/L NAA treatments (Supplementary Fig. S3A–D). Interestingly, sequencing results revealed 16 insertions and 10 deletions in 0.1 mg/L NAA treatment, compared to 9 insertions and 18 deletion events in 0.5 mg/L NAA treatment (Supplementary Fig. S3C). Additionally, two events with large deletions (>20 bp) were detected in the 0.5 mg/L NAA treatment, but not in the 0.1 mg/L NAA treatment (Supplementary Fig. S3D). These findings suggest that higher concentrations of auxin may promote more deletion activities, particularly large deletions. Further studies are required to elucidate the mechanisms underlying this observation.

In addition, an experiment was repeated using only a single gRNA under 0.1 and 0.5 mg/L NAA treatments (Supplementary Fig. S4A). The results showed that albino mutant efficiency was significantly higher in 0.5 mg/L NAA group, reaching 14.04%, which was 2.7 times higher than the 5.15% observed in the 0.1 mg/L NAA group. Additionally, the total visible mutant efficiency (chimeric and albino) increased from 11.34% in 0.1 mg/L NAA group to 20.34% in 0.5 mg/L NAA group. Notably, the chimera mutant efficiency remained relatively consistent between the two treatment groups (Supplementary Fig. S4B and C). These findings further indicate that higher auxin concentrations appear to enhance the generation of functionally edited plants in the T₀ generation.

### Morphological and histological differences in tobacco callus cells treated with varying concentrations of NAA

**Figure 3:**
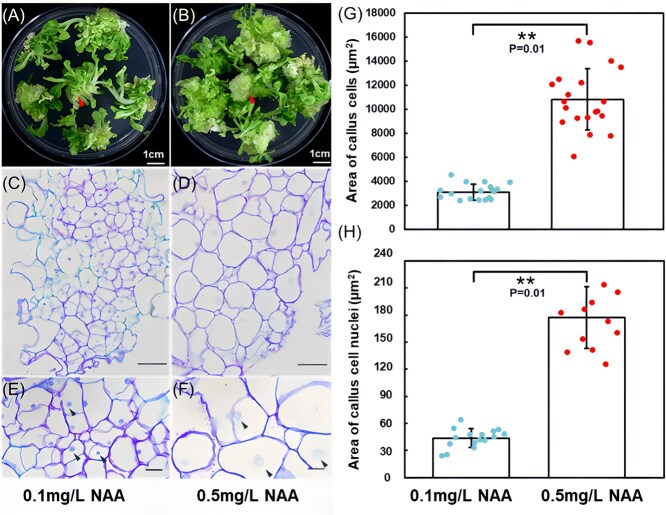
Morphological and histological observation of tobacco callus under 0.1 or 0.5 mg/L NAA treatments. (A and B) Morphological comparison of explants treated with 0.1 mg/L NAA (A) or 0.5 mg/L NAA (B) treatments after 51 days of culture, with the arrowheads indicating the calli selected for subsequent resin sectioning. (C) Histological observation of callus cells under 0.1 mg/L NAA treatment. (D) Histological observation of callus cells under 0.5 mg/L NAA treatment. (E) Amplified details from (C). (F) Amplified details from (D); The arrowheads in (E) and (F) highlight the stained nuclei. (G) Comparison of callus cell areas between 0.1 and 0.5 mg/L NAA treatments. (H) Comparison of callus nuclear areas between 0.1 and 0.5 mg/L NAA treatments. The calli used for the microscope observation were carefully excised from explants, ensuring no shoot tissues remained. Scale bar = 100 um. Error bars represent the standard error values, with asterisk denoting significant difference between different groups (Student’s *t*-test, *P* <0.01).

Our observations revealed distinct variations in callus development across different NAA concentrations. The smallest calli were generated in the 0.1 mg/L NAA group, while the largest originated from 0.5 mg/L NAA group (Fig. 2E and Supplementary Fig. S2). Consequently, calli from these two groups underwent further morphological and histological analysis. To eliminate potential interference from transformation and gene editing processes, we conducted tobacco tissue culture without *Agrobacterium* infection under the 0.1 and 0.5 mg/L NAA treatments. The regeneration progress was highly consistent with our previous experiment. At the early stage (25 days of culture), shoot initiation was observed in a few explants treated with 0.1 mg/L NAA, whereas no such initiation occurred in the 0.5 mg/L NAA treatment (Supplementary Fig. S5). By the full-grown stage (51 days of culture), calli from the 0.1 mg/L NAA treatment (Fig. 3A) were noticeably smaller than those from the 0.5 mg/L NAA treatment (Fig. 3B). Importantly, all regenerated shoots appeared healthy and green at this stage (Fig. 3A and B), suggesting that NAA treatment alone does not induce the albino phenotype.

Additionally, our histological analysis revealed profound cellular differences. Callus cell in the 0.5 mg/L NAA treatment (Fig. 3D and G) exhibited irregular arrangements and were approximately 2.5 times larger than those in the 0.1 mg/L NAA treatment (Fig. 3C and G). Correspondingly, the nuclei of callus cells from 0.5 mg/L NAA treatment were about 3.1 times larger than those from 0.1 mg/L NAA treatment and showed a relatively faint staining (Fig. 3F and H). In contrast, the nuclei of callus cells from 0.1 mg/L NAA treatment were readily stained and distinguishable, despite their much smaller size (Fig. 3E and H). These differences in cell and nuclei size, as well as staining intensity between 0.1 and 0.5 mg/L NAA treatments, were already detectable in various tobacco callus regions at the early stage (25 days of culture) (Supplementary Fig. S6) and became increasingly pronounced as the culture period extended.

### Altered expression of genes related to chromatin remodeling and plant regeneration in tobacco calli treated with different concentrations of NAA

**Figure 4:**
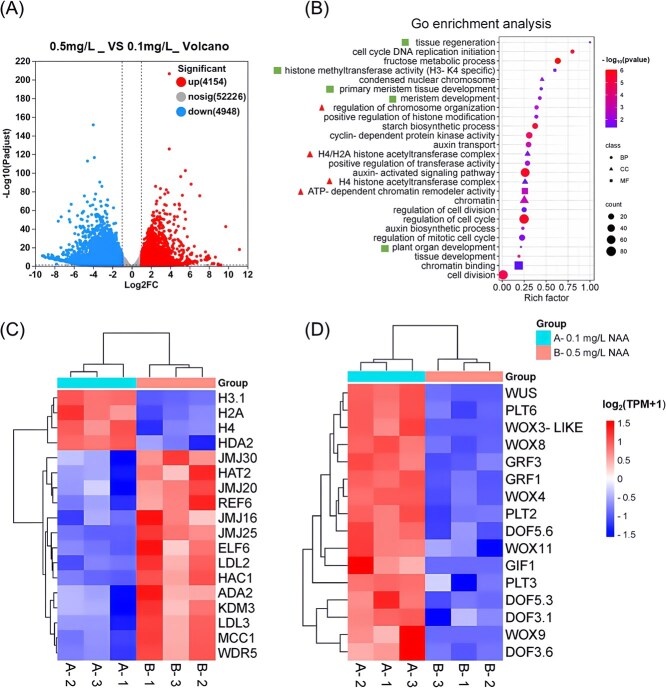
RNA-seq analysis of tobacco calli treated with 0.1 mg/L or 0.5 mg/L NAA for 25 days. (A) Total counts of up-regulated and down-regulated genes in the RNA-seq comparison. (B) Gene Ontology (GO) analysis highlighting significant enrichments in various category terms, with chromosome organization highlighted by the triangles and plant regeneration categories indicated by the squares. (C) Heat map of DEGs related to chromosome organization (Adjusted *P* <0.05, *n* = 3). (D) Heat map of DEGs related to plant regeneration (Adjusted *P* <0.05, *n* = 3).

**Figure 5:**
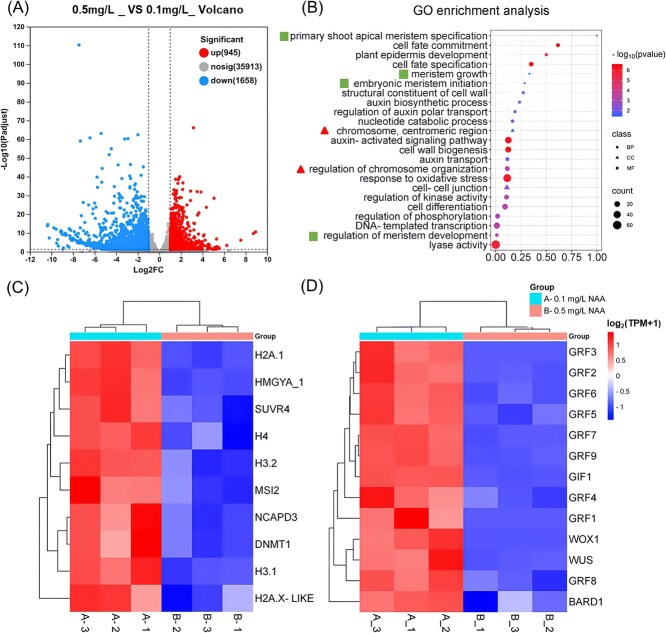
RNA-seq analysis of tobacco calli treated with 0.1 or 0.5 mg/L NAA for 51 days. (A) Total counts of up-regulated or down-regulated genes identified from the RNA-seq analysis. (B) GO term analysis showing significant enrichments in various category terms, with chromosome organization highlighted bythe triangles and plant regeneration categories indicated by the squares. (C) Heat map of DEGs related to chromosome organization (Adjusted *P* <0.05, *n* = 3). (D) Heat map of DEGs related to plant regeneration (Adjusted *P* <0.05, *n* = 3).

To identify the differentially expressed genes (DEGs) responsive to the higher auxin concentration, RNA-seq analysis was conducted on tobacco calli cultured with 0.1 and 0.5 mg/L NAA for 25 days (early stage; Supplementary Fig. S5) and 51 days (fully grown stage; Fig. 3A and B). Our findings revealed that the higher NAA (0.5 mg/L) significantly altered the expression of 9102 genes at the early stage (DEGs with |log2FC| ≥ 1 and *P*-adjust <0.05 were considered to be significantly differentially expressed), including 4154 significantly up-regulated and 4948 significantly down-regulated genes compared to the 0.1 mg/L treatment (Fig. 4A; *P*-adjust<0.05). At the fully grown stage, 2603 genes were differentially expressed, including 945 significantly up-regulated and 1658 significantly down-regulated genes under the 0.5 mg/L NAA treatment (Fig. 5A; *P*-adjust<0.05). Gene ontology (GO) analysis revealed enrichments in terms related to chromosome structure and chromatin dynamics (Figs 4B and 5B; red triangles). For instance, histone genes such as *H2A.1/X-like, H3.1,* and *H4* were significantly downregulated at both stages (Figs 4C and 5C). Additionally, genes associated with nucleosome assembly (*HMGYA_1, HMG-Y-related protein A*) and chromatin condensation (*NCAPD3, condensin-2 complex subunit D3*) were also significantly downregulated, though these effects were limited to the fully grown stage (Fig. 5C).

Conversely, genes involved in histone modifications, including demethylation and acetylation, were significantly upregulated at early stage in the 0.5 mg/L NAA treatment (Fig. 4C). Interestingly, numerous shoot regeneration-related genes, including *WUSCHELWUSCHEL* (*WUS*)*/WUSCHEL-RELATED HOMEOBOX* (*WOX*)*, PLETHORA* (*PLT*) and *GROWTH-REGULATING FACTOR* (*GRF*)*,* were significantly downregulated at both stages under the 0.5 mg/L NAA treatment compared to the 0.1 mg/L NAA treatment (Figs 4D and 5D), while *DNA-BINDING ONE FINGER* (*DOFs*) were affected only at the early stage. This downregulation of key shoot regeneration-related genes correlates with the observed delay and reduction in shoot formation in the 0.5 mg/L NAA treatment compared to the 0.1 mg/L NAA treatment (Figs 1A and 2D). The differentially expressed genes and corresponding gene loci in tobacco reference genome were shown in Supplementary Table S1.

### The effects of NAA concentrations on chromatin accessibility in tobacco cells

**Figure 6:**
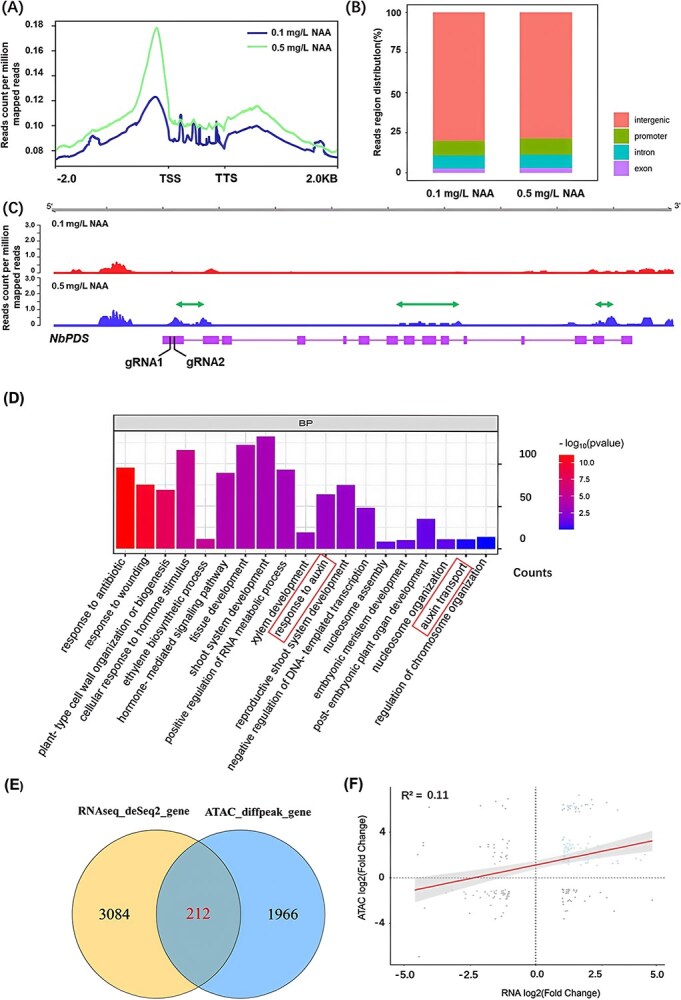
Effects of auxin on chromatin accessibility in tobacco cells. (A) Analysis of chromatin accessibility distribution using ATAC-seq in tobacco cells cultured on media with 0.1 or 0.5 mg/L NAA for two weeks. The graph shows the density of accessible chromatin regions from 2 kb upstream to 2 kb downstream of the gene body, including transcriptional start sites (TSS) and transcriptional termination sites (TTS). (B) Overview of genome-wide distribution of reads region, illustrating the landscape of chromatin openness across the entire genome. (C) Enrichment of ATAC-seq signals within the target gene *NbPDS3*, highlighting specific areas of increased accessibility marked by double arrowhead lines. The boxes represent the exons of *NbPDS3* gene, and two gRNAs targeting the first exon are indicated by the lines, the read count per million mapped reads reached 0.41 at the gRNA2 site, which was much higher than the 0.13 observed at gRNA1 site. (D) Genes with significantly different chromatin accessibility (0.5 vs 0.1 mg/L NAA) were used for the GO analysis, which revealed significant enrichments of genes across various categories, including auxin-related genes highlighted by the frames. (E) Overlapping Venn plot of genes with differential expression and genes with differential signals of chromatin accessibility. (F) Pearson correlation of the accessibility of the nearest genes versus the differential expression.

Chromatin accessibility, defined as the physical availability of genomic DNA to regulatory elements such as transcription factors, is closely associated with chromosome architecture and compactness [44]. In our study, more intense nuclear staining was observed in the tobacco callus cells treated with 0.1 mg/L NAA compared to those treated with 0.5 mg/L NAA, suggesting chromatin decondensation with increasing NAA concentration (Fig. 3E and F). This observation aligns with our RNA-seq results (Figs 4C and 5C), leading us to hypothesize that chromatin accessibility is influenced by different concentrations of NAA. To test this hypothesis, we assessed chromatin accessibility of tobacco cells treated with 0.1 or 0.5 mg/L NAA for two weeks using ATAC-Seq. ATAC-Seq identifies accessible chromatin through sequencing and mapping DNA fragments generated by Tn5 transposase, which simultaneously cleaves DNA and ligates sequencing adapters to the open chromatin regions [45]. Our findings confirmed that higher auxin levels (0.5 mg/L NAA) increased chromatin accessibility in tobacco cells (Fig. 6A). Despite this increase, the distribution patterns of accessible chromatin regions were similar between 0.1 and 0.5 mg/L NAA treatments, with sequencing reads predominantly mapping to intergenic regions, followed by promoter and intron regions, and least in exon regions (Fig. 6B). Additionally, the analysis of ATAC-seq signal enrichment in the target gene *NbPDS3* revealed greater chromatin accessibility in the 0.5 mg/L NAA treatment compared to the 0.1 mg/L NAA treatment as indicated by green double arrowhead lines (Fig. 6C). Both gRNAs targeted the first exon of *NbPDS3*, with their positions marked by black lines in Fig. 6C. Notably, the region targeted by gRNA2 showed significantly higher chromatin accessibility compared to the region targeted by gRNA1, as indicated by the strong ATAC-seq signal at the gRNA2 site (Fig. 6C and Supplementary Fig. S1A). Following the higher auxin treatment, the read count per million mapped reads reached 0.41 at the gRNA2 site, which was much higher than the 0.13 observed at gRNA1 site. This difference in chromatin accessibility correlates with the higher frequency of gene editing events at the gRNA2 site. Sequencing results of 25 samples each from both 0.1 mg/L and 0.5 mg/L NAA treatments are shown in Supplementary Fig. S3A and B. In 0.1 mg/L NAA treatment, 19 gene-edited events were detected at the gRNA2 target site, while only 7 gene-edited events at gRNA1 sites; Similarly in 0.5 mg/L NAA treatment, 22 gene-edited events were detected at the gRNA2 target site, while only 5 gene-edited events at gRNA1 sites.

In addition, genes with significantly different chromatin accessibility (0.5 vs 0.1 mg/L NAA) were used for the GO analysis. The results revealed significant enrichment of genes across various categories, including auxin-related genes, which were highlighted by red frames in Fig. 6D. Additionally, RNA-seq analysis was conducted on the same batch of explants used for ATAC-seq, identified 212 genes with transcriptional changes associated with differentially accessible chromatin regions (Fig. 6E). While enhanced chromatin accessibility generally correlated with increased transcription, the correlation coefficient (*R*^2^ = 0.11) was relatively low (Fig. 6F). A subset of regions exhibited decreased accessibility but increased transcription, suggesting the complex regulatory mechanisms governing genes expression.

To further investigate the regulatory mechanisms underlying auxin-induced transcriptional changes, we performed *in silico* transcription factor binding site (TFBS) analysis to explore the presence and distribution of canonical auxin-responsive *cis*-elements within the promoter regions (2 kb upstream) of selected differentially expressed genes (DEGs) associated with plant regeneration and chromatin regulation (Supplementary file 1. RNA-seq align with ATAC-seq). Using PlantCARE and PlantPAN, we identified five canonical auxin-responsive motifs: TGA-element (AACGAC), AuxRR-core motif (GGTCCAT), AuxRE-ARF (TGTCTC), GH3 motif (CATATG), and SAUR motif (CACATG), which are known binding sites for auxin-responsive transcription factors, including ARFs and Aux/IAA-interacting partners (Supplementary Fig. S7A). The number and type of auxin-responsive motifs varied present in these analyzed genes. For instance, *Growth Regulating Factor 1* (*GRF1*) and *a Jumonji-domain–containing histone demethylase* (*JMJ25*) each harbored three TGA-elements and one AuxRE or SAUR motif. In contrast, chromatin remodelers like *KDM3(LYSINE DEMETHYLASE 3)* and *histone acetyltransferase 1* (*HAC1*) and *Growth Regulating Factor 3* (*GRF3*) each contained a single TGA-element. *WUSCHEL-RELATED HOMEOBOX 1* (*WOX1*) and *DNA-BINDING ONE FINGER 5.3*(*DOF5.3*), two transcription factors implicated in organogenesis, displayed a combination of three different kinds of auxin-responsive motifs, suggesting possible integration of multiple hormonal signaling pathways at their promoters. Notably, *SET-DOMAIN CONTAINING PROTEIN LYSINE METHYLTRANSFERASE 4* (*SUVR4*), contained the highest density of auxin-related motifs, including two SAUR, one GH3, one AuxRE, and one TGA element.

We next integrated these findings with chromatin accessibility data derived from ATAC-seq to examine whether auxin concentration influenced DNA openness in cis-regulatory regions near these motifs. This analysis revealed four representative regulatory genes, *WOX1, SUVR4, KDM3, and JMJ25*, that exhibited pronounced changes in chromatin accessibility at regions harboring TGA-elements, GH3 and/or SAUR motifs (Supplementary Fig. S7B). Under high NAA treatment (0.5 mg/L), *WOX1* and *SUVR4* showed reduced chromatin accessibility near their TGA and GH3 motif loci, consistent with their transcriptional downregulation. In contrast, *KDM3* and *JMJ25* showed enhanced chromatin accessibility around both TGA and/or SAUR-containing promoter regions. Notably, *JMJ25* displayed a particularly strong accessibility gain at the promoter region containing three clustered TGA-elements. The increased accessibility in both genes aligned with their upregulated expression in response to elevated auxin levels (Supplementary Fig. S7B and Supplementary file 1 RNA-seq align with ATAC-Seq). These results suggest that auxin-responsive TFBS are functionally associated with chromatin remodeling events in both developmental and epigenetic regulatory genes. This integrative analysis further supports our mechanistic model in which exogenous auxin modulates both transcriptional activity and chromatin structure to enhance editing efficiency.

### Increased auxin concentration improved gene editing efficiency in tomato

Our results confirmed that a moderate increase in auxin concentration enhances the generation of functionally edited plants in the T₀ generation on tobacco. To assess the broad applicability of this method, we applied the same approach to knock out the *SlPDS* homologous gene in tomatoes. However, efficient plant regeneration, a critical prerequisite for successful gene editing, exhibited less tolerance to variations in auxin application in tomatoes. Our preliminary experiments revealed that continuous culture under high levels of auxin resulted in a much lower tomato shoot regeneration, although callus growth was promoted as NAA concentrations increased (Supplementary Fig. S8).

To mitigate the inhibitory effects of excessive auxin on shoot regeneration, we adopted a ‘two-phase’ culturing strategy for tomato explants. Initially, tomato cotyledons were cultured on tomato callus induction medium containing a series of NAA concentrations (50 150, 300 μg/L) combined with 2 mg/L Zeatin (ZT) for the first four weeks to induce the callus growth after *A. tumefaciens* inoculation. Then, all healthy explants were transferred to tomato shoot induction medium consisting of 20 μg/L NAA and 0.5 mg/L ZT to promote shoot regeneration.

**Figure 7:**
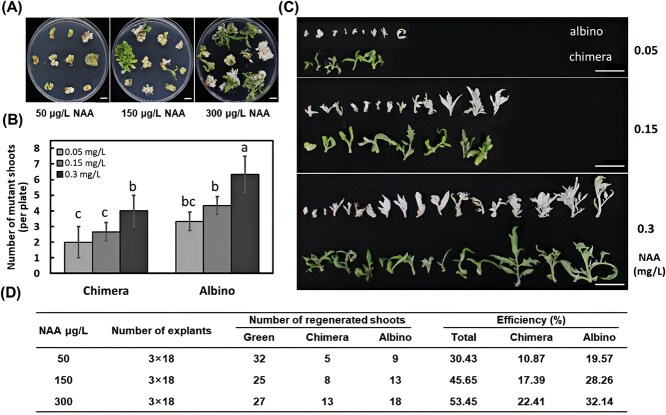
Effects of the pretreatment of explants with different concentrations of NAA on shoot regeneration and homozygous targeted gene editing in tomatoes. (A) Representation of tomato explants after 4 weeks of culture on shoot-induction media, following an initial four weeks of pretreatment with varying concentrations of NAA (50, 150, 300 μg/L). (B) Quantitative comparison of mutant shoots per petri dish across different concentrations of NAA pretreatment groups, including chimera (left) and complete albino shoots (right). (C) Visual comparison of chimera (bottom) and albino shoots (top) harvested from explants after seven weeks of culture on shoot-induction media, following pretreatment with different NAA concentrations. (D) Total numbers of regenerated shoots and mutant shoots derived from explants pretreated with different concentrations of NAA, along with corresponding efficiencies. Three replicates were applied to each treatment with each replicate consisting of 18 explants (cotyledons). Error bars represent the standard error values, with significant difference denoted by letters among different groups (ANOVA-LSD, *P* < 0.05). Scale bar = 1.5 cm in A and C.

An overview image of plant regeneration after four weeks of culture on shoot-induction media was shown in Fig. 7A. After an additional three weeks of culture, mutants from different groups were recorded, and corresponding gene editing efficiencies were calculated. A significantly higher number of mutant shoots (chimeric and albino) was observed in the 300 μg/L NAA pretreatment group (Fig. 7B). Representative mutant shoots were excised from calli for visual display (Fig. 7C). Specifically, the group pretreated with 300 μg/L NAA yielded the highest number of visible mutants −13 chimeric and 18 albino mutants, compared to 8 chimeric and 13 albino mutants from 150 μg/L NAA pretreatment group. By contrast, the 50 μg/L NAA pretreatment group produced the fewest mutants, with only 5 chimeric and 9 albino mutants (Fig. 7C and D). As a result, the highest total visible gene editing efficiency was recorded at 53.45% in the 300 μg/L NAA pretreatment group, comprising 22.41% chimeric and 32.14% albino mutants (Fig. 7D). In comparison, the 150 μg/L NAA group demonstrated a total efficiency of 45.65%, with 17.39% chimeric and 28.26% albino mutants. The lowest efficiencies were observed in the 50 μg/L NAA group, with a total of 30.43%, comprising 10.87% chimeric and 19.57% albino mutants (Fig. 7D). In conclusion, the results confirmed that the pretreatment with higher auxin concentrations during the first four weeks of culture improved tomato gene editing efficiency without compromising subsequent shoot regeneration when explants were transferred to an optimal shoot-induction medium (Fig. 7D). Gene editing events, including albino or chimeric shoots, were validated through PCR sequencing, with insertions or deletions detected in the representative samples (Supplementary Fig. S9). The PCR primers used for detecting gene-edited events were included in Supplementary Table S2.

### Detection of off-target gene-edited mutations in tobacco and tomato

To investigate whether higher concentrations of auxin promote off-target gene editing events, three albino tobacco shoot samples were collected from each of the 0.1 and 0.5 mg/L NAA treatments. The three most likely off-target loci, as predicted by CRISPOR [46], were amplified using the specific primers (Supplementary Table S3) and analyzed by sequencing. The sequencing results showed no detectable off-target mutations at these loci (Supplementary Fig. S10A and B).

Similarly in tomato, off-target effects were assessed using three albino shoot samples from 50, 150, and 300 μg/L NAA treatments, respectively. Sequencing analysis of the three most likely off-target loci detected no mutations in any of the examined regions (Supplementary Table S4 and Supplementary Fig. S11A and B). While these results suggest high targeting specificity under our experimental conditions, we acknowledge that additional off-target effects might exist beyond the scope of our current detection methods.

### Detection of the effect of auxin on the Cas9 activity *in vitro*

To investigate this possibility, we performed an *in vitro* cleavage assay using Cas9 in vitro Cleavage Kit (PC1400, Inovogen Tech. Co., Beijing, China). The assay utilized a 760-bp positive control DNA containing a single Cas9 target site and the corresponding sgRNA. After incubation with Cas9, successful cleavage will generate two fragments of 310 and 450 bp, as shown in Supplementary Fig. S12A. Our results demonstrated no difference in Cas9 cleavage activity with or without auxin treatment for 10, 20, and 30 min (Supplementary Fig. S12A). For quantitative comparison, we measured the DNA band intensities from 10 min cleavage reactions by using ImageJ [47]. The result showed no significant difference in the percentage of cleaved products between control (48.75 ± 1.90%) and the 0.5 mg/L NAA treatment (49.53 ± 3.26%) (Supplementary Fig. S12B). These results clearly demonstrate that auxin treatment does not enhance Cas9 cleavage activity *in vitro*.

## Discussion

Plant regeneration was governed by the dynamic interplay between auxin and cytokinin. It is well established that a higher auxin to cytokinin ratio typically promotes callus formation *in vitro*, while lower ratio favors shoot regeneration [48–50]. This principle aligns with our observations in tobacco and tomato regeneration, where an elevated auxin to cytokinin ratio by applying higher NAA without altering cytokinin levels significantly enhanced callus size *in vitro* (Figs 2E and 3B). Our RNA-seq analysis revealed that increasing NAA concentration from 0.1 to 0.5 mg/L during *in vitro* culture led to the downregulation of key genes involved in cell pluripotency at the early callus growth stage. These genes include *WUS/WOX, PLT, DOF,* and *GRF* [51–55]. Interestingly, we also noticed that genes associated with plant regeneration, particularly those in the *GRF* family, which encode plant-specific transcription factors critical for plant development [56, 57], were predominantly downregulated at the full-grown stage (Fig. 5D). This finding corroborates previous studies that highlight the crucial role of *GRFs* in shoot regeneration [58–60]. Our results suggested that different auxin to cytokinin ratios may affect shoot regeneration through altering the expression of these key developmental genes that govern plant regeneration during *in vitro* tissue culture. The reduced expression of these genes at higher auxin concentration (i.e. 0.5 mg/L NAA) leads to decreased and delayed tobacco shoot formation compared to a lower one (i.e. 0.1 mg/L NAA) (Figs 1A and 2D).

Tobacco, renowned for its exceptional plant regeneration capabilities, exhibited resilience to varying concentration of NAA, showing no significant inhibition in shoot formation until the NAA concentration reached 0.7 mg/L in our study (Fig. 2D). However, the optimal phytohormone balance is crucial for successful plant regeneration across most plant species, particularly for those with limited regeneration capacity. For example, a combination of 50 μg/L NAA with 2 mg/L ZT is ideal for tomato shoot regeneration, but an increase to 300 μg/L NAA in media significantly reduced shoot formation (Supplementary Fig. S8). Considering the crucial role of plant regeneration in generating gene-edited plants, we adopted a ‘two-phase’ culture strategy to mitigate the detrimental effects of excessive auxin on tomato plant regeneration. Our results suggested that using higher auxin concentrations during the callus induction phase successfully enhanced targeted gene editing efficiency without compromising subsequent shoot regeneration in tomatoes (Fig. 7D).

Auxin, a central regulator of plant development, exerts concentration-dependent effects on cell division, elongation, and differentiation, with outcomes further influenced by developmental stage and species-specific sensitivity [61–63]. While it is true that certain cellular responses—such as callus proliferation or changes in nuclear architecture—may arise from treatments with other phytohormones (e.g. cytokinin) due to convergent effects on cell cycle regulation and chromatin remodeling [62, 64, 65], the specific combination of morphological changes and enhanced genome editing efficiency observed in our study appears to be predominantly driven by exogenous auxin application in the tobacco system. The induction of a highly proliferating, undifferentiated callus by a specific auxin concentration is a well-established prerequisite for successful genetic manipulation in various plant species, including tobacco [53]. Therefore, although the effects described here reflect auxin-specific responses in our tobacco tissue culture, their universality across hormone classes and diverse plant species remain an important area for future comparative studies.

In our study, increasing NAA from 0.1 to 0.5 mg/L led to enlarged cells and nuclei in tobacco calli (Fig. 3G). Interestingly, nuclei in callus cells treated with 0.1 mg/L NAA displayed intense staining, whereas those treated with 0.5 mg/L NAA exhibited fainter staining (Fig. 3C–F). This suggests a relaxation in chromatin structure at the higher NAA concentration, as condensed chromatin (heterochromatin) is known to stain more intensely than less condensed chromatin (euchromatin) [66, 67]. Given that a relaxed chromatin structure is associated with enhanced chromatin accessibility [68, 69], we hypothesized that chromatin accessibility was improved in cells treated with higher NAA concentration. This hypothesis was confirmed by our ATAC-seq results, which detected a higher density of sequencing reads in gene body regions in tobacco cells treated with 0.5 mg/L NAA compared to those treated with 0.1 mg/L NAA (Fig. 6A).

In eukaryotic species, genomic DNA is wrapped around a core histone octamer to form a nucleosome, which is further folded into functional genome units called chromatin [70]. Notably, nucleosomes have been shown to impede Cas9 access to DNA, yet this obstruction can be mitigated by chromatin remodeling [71, 72]. Therefore, the observed decrease in the expression of histone genes, nucleosome assembly related genes and chromatin condensation-related genes under 0.5 mg/L NAA treatment (Figs 4C and 5C) likely contributes to the increased chromatin accessibility and gene editing efficiency. Additionally, the upregulation of the genes related to histone demethylation/acetylation, which is known to increase chromatin accessibility by remodeling chromatin structure [73, 74], was also particularly evident at the early callus growth stage under 0.5 mg/L NAA treatment (Fig. 4C). Furthermore, through *in silico* promoter analysis, we screened the differentially expressed genes that have been identified for their roles in regeneration or epigenetic regulation and found that sixteen genes contained at least one auxin-responsive cis-element (Supplementary Fig. S7A). Among these, four genes, *WOX1*, *SUVR4*, *KDM3*, and *JMJ25*, exhibited notable changes in chromatin accessibility in the promoter regions surrounding auxin-responsive *cis*-elements (Supplementary Fig. S7B). *WOX1* is a key developmental transcription factor involved in apical meristem maintenance, lateral organ formation, and hormone signaling [75]. *SUVR4* functions as a histone methyltransferase, while *KDM3* and *JMJ25* are histone demethylases implicated in chromatin remodeling and transcriptional reprogramming [76–78]. The observed auxin-associated chromatin accessibility shifts in these loci further support our hypothesis that exogenous auxin enhances genome editing efficiency by modulating both regeneration-related gene expression and chromatin openness at epigenetic regulators. While these findings strengthen our mechanistic model, the precise signaling cascade through which auxin coordinates transcription factor activity and chromatin remodeling remains to be elucidated. Future studies should investigate how auxin-responsive elements are functionally interpreted by specific transcription factors and chromatin modifiers to establish an editing-permissive cellular state.

A recent study showed that switching explants from a callus induction media with higher auxin to a shoot induction media containing higher cytokinin caused dynamic changes in chromatin accessibility of different genes at various timing points [79]. Unlike that study, our comparison focused on the chromatin accessibility changes triggered by different concentrations of auxin at the same timing point. Further investigation may be needed to explore dynamic changes in chromatin accessibility under different concentrations of exogenous auxin at various timing points. In addition to exogenous hormones, chromatin accessibility can also be significantly affected by environmental cues. For example, Tian, et al. [80] found that a long day photoperiod can greatly increase chromatin accessibility in vascular companion cells of *A. thaliana*. Similarly, Wang et al. [81] found low temperatures significantly changed chromatin accessibility across the genome of tea plants. These findings suggested that plant genomes undergo global changes in response to external factors.

In this study, we efficiently improved gene editing efficiency through enhancing the chromatin accessibility by modifying auxin concentrations during *in vitro* tissue culture. Similarly, several studies have demonstrated that applying histone deacetylase inhibitors (HDACis) in the culture media can increase chromatin accessibility, thereby improving plant genetic transformation and gene editing efficiency [28–30]. In addition to enhancing spatial accessibility for Cas9 to function, the higher NAA concentration prolonged the growth duration for dedifferentiated callus cells and delayed shoot initiation (Fig. 1A). This may provide an extended timing window for Cas9 to bind and edit the target gene during the callus growth stage, which could partly explain the higher incidence of albino editing events in the 0.5 mg/L NAA group (Fig. 2D and Supplementary Fig. S4C). By contrast, chimerism may be caused by uneven editing events that occur in differentiated cells during shoot initiation and growth. This finding indicates that achieving uniform editing in dedifferentiated cells is crucial for producing functionally edited plants in T_0_ generation.

In this study, we observed a significantly higher albino mutation efficiency following higher auxin treatment in tobacco. In our initial experiment using two guide RNAs, we identified 10 albino mutants out of 156 regenerated shoots (6.41%) in the 0.1 mg/L NAA treatment group, compared to 17 albino mutants from 112 regenerated shoots (15.18%) in the 0.5 mg/L NAA treatment group (Fig. 2D). To further confirm our findings, we repeated the experiment using a single gRNA with the same NAA concentrations (0.1 and 0.5 mg/L) (Supplementary Fig. S4A). The results were consistent with our initial findings: the albino mutation efficiency in the 0.5 mg/L NAA group (14.04%) was approximately 2.7 times higher than in the 0.1 mg/L NAA group (5.15%) (Supplementary Fig. S4B and C). Therefore, we concluded that higher auxin treatment significantly enhances the generation of functionally edited plants in the T₀ generation in tobacco. This effect appears to be independent of the number of gRNAs used in the gene editing system. While the albino phenotype strongly suggests complete functional disruption of the *PDS3* locus, comprehensive sequencing analysis would be required to distinguish true homozygous mutations from compound biallelic or multi-allelic outcomes. This distinction, while mechanistically interesting, does not diminish the practical utility of our approach for generating functionally edited plants in the T₀ generation.

We investigated potential off-target effects using Sanger sequencing and found no off-target events in the predicted regions. This observation aligns with previous studies that reported a low frequency of off-target mutagenesis in CRISPR/Cas9 applications in plants [82, 83]. However, we acknowledge that limitations in our detection methods prevented us from completely ruling out all possible off-target effects. Recent studies have demonstrated various approaches to enhance CRISPR/Cas9 editing efficiency while maintaining specificity. Liu et al. [28] found that inhibiting histone deacetylase 1 increased chromatin accessibility and improved genome editing efficiency in human cells without detecting off-target events. Similarly, in rice, Liu et al. [25] achieved enhanced gene editing efficiency without increased off-target activity by fusing a synthetic transcription activation domain and proximal dsgRNAs to Cas9. In contrast, LeBlanc et al. [47] found that short-time heat stress increased both on-target and off-target mutagenesis in *Arabiposis* through modulating Cas9 activity, although they demonstrated that two nucleotide substitutions within the gRNAs were sufficient to prevent off-target mutagenesis. In our study, we observed that there are more than three mismatches between the on-target gRNAs and potential off-target sites in both tobacco and tomato (Supplementary Figs S10A and S11A). This likely explains why no off-target mutations were detected, even with the increased mutation efficiency resulting from enhanced chromatin accessibility (Supplementary Figs S10B and S11B). These findings emphasize the critical importance of designing gRNAs with high specificity in CRISPR applications, particularly when employing strategies to increase mutation efficiency.

The enhanced generation of functionally edited plants in the T₀ generation has significant implications for accelerating breeding process, especially in polyploid species with long growth cycles. This improvement is especially valuable when combined with transgene-free genome editing technology, as it addresses key challenges in plant species with complex genetic backgrounds and/or asexual reproduction modes, such as fruit trees. The ability to generate gene editing events with mutator phenotype in the T_0_ generation eliminates the need for advancing heterozygous mutations through seed segregation, thereby preserving desirable traits and facilitating the acquisition of transgene-free mutants [84, 85]. It is noteworthy that the effect of auxin on heterozygous or mosaic mutation efficiency was not assessed in this study. Given that heterozygous individuals often lack visible phenotypes, especially in *PDS3*-edited lines, additional sequencing-based analyses would be required to address this. While we hypothesize that these outcomes may also improve under auxin treatment, this remains to be validated experimentally.

In addition, Higher auxin treatment may not uniformly enhance chromatin accessibility across all genomic regions. If target sequences are located in regions where chromatin accessibility is unaffected or negatively impacted by auxin, gene editing efficiency may not be improved as expected. Therefore, using multiple gRNAs targeting on different regions could increase the likelihood of successful editing. The differential enhancement of different gRNAs in response to varying auxin concentrations may partly reflect auxin signaling-mediated modulation of chromatin accessibility at specific genomic loci. Recent studies have shown that auxin signaling can indeed influence chromatin states, often in a context-specific manner. For example, the auxin-responsive transcription factor *ARF5 (MONOPTEROS)* has been implicated in modulating nucleosome occupancy at developmental gene targets, potentially through recruitment of chromatin remodelers such as *BRAHMA* from the SWI/SNF complex [86]. In parallel, Aux/IAA repressors recruit co-repressor complexes such as *TOPLESS (TPL)* and associated histone deacetylases (HDACs) to suppress transcription at auxin-responsive loci [87]. These findings support the possibility that different components of the auxin signaling network can differentially modulate chromatin architecture, with consequences for CRISPR activity that merit further investigation.

Additionally, it should be acknowledged the limitations of our findings: (i) the effects of auxin on chromatin accessibility and regeneration may vary significantly across species and genotypes. In recalcitrant crops or those with lower developmental plasticity, such as monocots or perennials, auxin signaling may function differently, potentially limiting the direct applicability of findings derived from a permissive model like tobacco. Further comparative studies are needed to validate this approach across diverse plant systems. (ii) Our findings are based on stable transformation systems. In transient (e.g. geminivirus-based) or DNA-free delivery platforms, Cas9 and gRNA are not constitutively expressed, thereby narrowing the temporal window for auxin to exert its full chromatin-modulating effects. As such, the promotive influence of auxin on editing efficiency may not fully translate to these systems. (iii) While elevated auxin levels appear to enhance gene editing efficiency and are associated with increased chromatin accessibility at key regulatory genes, the precise mechanism through which auxin signaling influences chromatin states during tissue culture remains unclear. Future studies will be necessary to elucidate how auxin-responsive pathways modulate chromatin dynamics and regeneration competence.

## Conclusions

**Figure 8:**
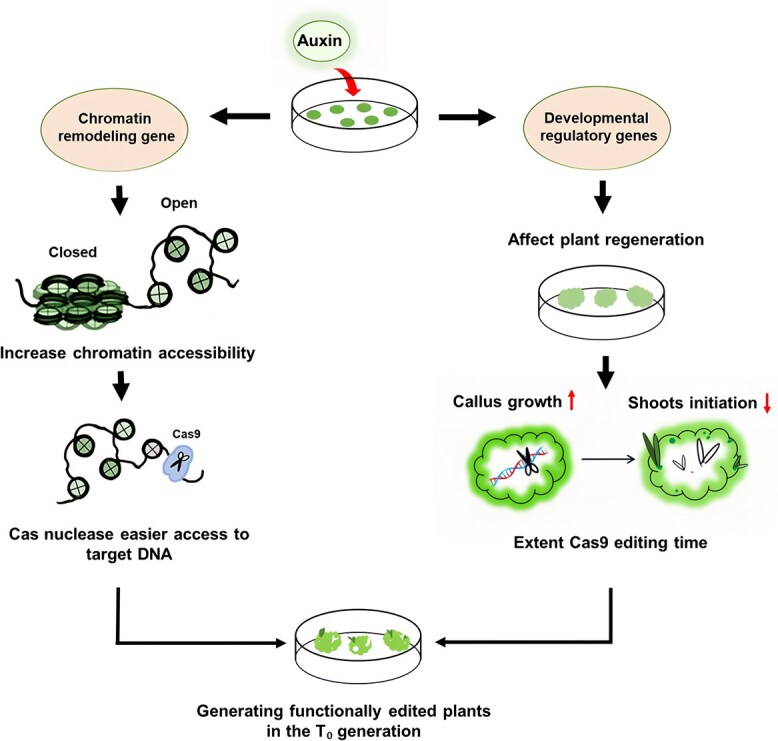
Schematic diagram illustrating the effects of exogenous auxin application during *in vitro* tissue culture on generation of functionally edited plants in the T₀ generation. On the left, auxin increases chromatin accessibility within the plant genome, facilitating Cas9 access to DNA and enhancing editing efficiency. On the right, auxin improves gene editing efficiency by promoting callus growth and delaying shoots initiation, which provide Cas9 additional time to function effectively in dedifferentiated callus cells before shoots formation.

Our study has revealed new aspects of auxin function in affecting gene editing efficiency in plants as shown in schematic diagram (Fig. 8). First, the higher auxin concentration affects chromatin states, enabling Cas9 efficiently access to the genomic DNA regions with improved chromatin accessibility. On the other hand, increased auxin concentration promotes calli growth and delays shoots initiation, providing a longer timing window for Cas9 protein to exert its function before shoot regeneration, thus enhance generation of functionally edited plants in the T₀ generation. Based on this model, a ‘two-phase’ culture strategy was developed to overcome the inhibitory effect of excessive auxin application on shoot regeneration, which may be used to accelerate the progress of gene editing breeding in various plant species.

## Materials and methods

### Tissue culture media

The basal Murashige and Skoog (MS) medium was used for tissue culture, consisting of 4.4 g/L MS salts and vitamins (Phyto Technology Laboratories, USA), 30 g/L sucrose, and 8 g/L agar. The pH of the medium was adjusted to 5.8 before autoclaving at 121°C for 25 min. Stock solutions of hormones were filter-sterilized and added to the autoclaved medium when it cooled to 50°C. The following media were used in this study for different purposes: preculture medium: MS without hormones and antibiotics. Tobacco callus and shoot induction medium: MS + 2 mg/L 6-BA+100 mg/L kanamycin+100 mg/L Timentin+ varying concentrations of NAA (0.1, 0.3, 0.5, and 0.7 mg/L). Tomato callus induction medium: MS + 2 mg/L ZT + 100 mg/L kanamycin+100 mg/L Timentin+ varying concentrations of NAA (50, 150, 300 μg/L). Tomato shoot induction medium: MS+ 20 μg/L NAA + 0.5 mg/L ZT + 100 mg/L kanamycin+100 mg/L Timentin.

### Transformation and tissue culture procedure

Seeds of tobacco (*N. benthamiana*) and tomato (*Solanum lycopersicum* cv. Alisa Craig) were produced in our lab. Seeds were first surface-sterilized with 70% ethanol for 90 seconds, followed by three times of rinsing with deionized H_2_O; these seeds were then treated with 15% bleach for 20 min, and subsequently washed five times with ddH_2_O and placed on MS medium for germination. Fully extended cotyledons from seedlings were excised and cut into approximately 0.5 × 0.5 cm^2^ pieces. These explants were precultured in the dark at 25°C for 2 days prior to transformation.

A 20 μl *A. tumefaciens* stock solution was initially cultured overnight in 5 ml liquid LB medium (Thermo Scientific, USA) at 28°C, with 180 rpm shaking. Subsequently, 1 ml of this culture was used to inoculate 10 ml fresh LB medium for a second round of culture until the O.D.600 reached 0.5. *A. tumefaciens* cells were collected by a centrifugation at 4000 g for 15 min and resuspended in liquid MS (4.4 g/L MS salts and vitamins, 2% sucrose, pH of 5.8) to O.D.600 of 0.8. Cotyledon explants were immersed in the *A. tumefaciens* suspension for 15 min, and then placed back to the preculture MS medium for two days in dark conditions at 25°C. Following co-cultivation, all infected tobacco explants were directly transferred to tobacco callus and shoot induction medium. The explants were subjected to regular subculture approximately every 4 weeks for 51 days. Tomato explants were firstly placed on tomato callus induction medium for the first four weeks, followed by transferring to tomato shoot induction medium. All cultures were maintained under a 16 h/8 h light/dark cycle at 25°C. Callus development and shoot regeneration were photographically recorded.

### Vector construction

The gene editing system employed in this study was modified from the previously reported PHN-SpCas9-4xBsaI-GFP vector [88]. The vector contains a plant codon-optimized SpCas9 gene driven by a parsley ubiquitin promoter and a NPTII gene under the control of a double-enhanced CsVMV (dCsVMV) promoter. Two guide RNAs (gRNAs) were designed using the web-based tools CRISPR-P (http://crispr.hzau.edu.cn/CRISPR2/) and CRISPOR (http://crispor.tefor.net/), which were selected for high predicted editing efficiency and minimal off-target potential. The gRNA sequences of CAGCTTATCTTTGGAGCTCGagg/ ACTCCATGGGGCATAAGTTAagg for *NbPDS3* (*Niben101Scf01283g02002*.1), and TTGGGAACTGAAAGTCGAGAtgg/GGCATGCAAAGTCTCTCAGGagg for *SlPDS* (*NC_015440.3:72003341–72 011 020*) were shown in the Supplementary Figs S1A and S9A. The selected gRNAs were synthesized by Gene Universal Inc. (Newark, DE) and cloned into the PHN-SpCas9-4xBsaI-GFP vector at *BsaI* restriction sites. The gRNA was expressed under the control of the *Arabidopsis* AtU6 promoter and fused with a tRNA scaffold [89]. All the plasmid DNAs were transformed into *A. tumefaciens* strain EHA105 through freeze and thaw methods for subsequent plant transformation.

### PCR analysis and gene sequencing

Albino tobacco shoots regenerated from the calli cultured with either 0.1 or 0.5 mg/L NAA were collected for analysis. Genomic DNA was extracted from leaf tissues using the CTAB method [90]. To detect gene editing events, PCR amplification were performed with primers for two gRNA target sites using Q5 Hot Start High-Fidelity DNA polymerase (New England BioLabs, USA) following the manufacturer’s instructions. The amplified DNA fragments were separated by electrophoresis on a 1.2% (w/v) agarose gel, stained with SYBR green (Thermo Scientific, USA) and visualized using the Omega LumTM Imaging system (Gel Company, USA). The PCR products were subsequently purified for downstream analysis. The primers were used for detecting gene editing events were shown in Supplementary Table S2.

To assess potential off-target mutations, three tobacco albino shoots from each NAA treatment group (0.1 and 0.5 mg/L) were selected for PCR sequencing. The most likely off-target loci were identified using CRISPOR (Supplementary Fig. S10A) [46]. Primers for these regions were designed for PCR and Sanger sequencing (Supplementary Table S3). Similarly, in tomato, off-target effects were analyzed using three albino shoots from each of 50, 150, and 300 μg/L NAA treatments, respectively. The most likely off-target loci were shown in Supplementary Fig. S11A, and primers were listed in Supplementary Table S4. The mutation detection analysis was conducted with Synthego (https://ice.synthego.com/#/).

### RNA extraction and RNA-seq analysis

To determine transcript levels of tobacco calli treated with 0.1 or 0.5 mg/L NAA at 25 and 51 dpi, RNA extraction, purification and library construction were performed with Illumina® Stranded mRNA Prep, ligation kits according to the manufacturer’s instructions (Illumina, San Diego, CA, USA). The prepared libraries were quantified using a Qubit 4.0 fluorometer and sequenced on the Illumina NovaSeq 6000 platform, with three biological replicates applied for each treatment. The raw paired end reads were trimmed and quality controlled by fastp with default parameters. Clean reads were aligned to reference genome using HISAT2 software with orientation mode [91]. The mapped reads were assembled using StringTie in a reference-based approach. Differential expressed genes (DEGs) were identified using DESeq2, with gene expression levels calculated as transcripts per million reads (TPM) using RSEM. DEGs with |log2FC|≧1 & *P*-adjust <0.05 were considered to be significantly differentially expressed. GO analysis was performed by Goatools [92] (github.com/tanghaibao/GOatools), focusing on DEGs significantly enriched in distinct GO terms (*P*-adjust <0.05). The tobacco reference genome Niben261(solgenomics.net/organism/Nicotiana_benthamiana/genome) was used for analysis. The RNA-seq data have been deposited in the NCBI Sequence Read Archive (SRA) database under the accession code SRP500179, with the BioProject number PRJNA1097301. Detailed RNA-seq results are available in Supplementary File 1. RNA-Seq 25dpi and RNA-seq 51dpi.

### ATAC-Seq experiment

Approximately 80 tobacco explants were harvested after 2 weeks cultured on media containing either 0.1 or 0.5 mg/L NAA for nuclei isolation. The ATAC-seq was performed according to the guidelines of the ATAC-Seq Library Prep Kit for Illumina®. The paired-end sequencing of prepared DNA libraries was performed on the Illumina Novoseq 6000 platform. The clean sequencing data were aligned to the genome of the *Nicotiana_benthamiana* using BWA with default parameters (v0.7.17-r1188) [93]. All reads within 2 kb upstream and downstream of the transcriptional start site (TSS) and transcriptional termination site (TTS) were counted using deepTools (v3.5.12.0) [94]. The peaks and accessible chromatin region with *q*-value <0.05 were identified using MACS2 (v2.2.7.1) [95]. The different chromatin accessibility of genes (0.5 vs 0.1 mg/L) with |log2FC|≧2 & *P* < 0.05 were considered to be significantly different, which were screened by using FeatureCounts (v2.0.1) and edgeR (v3.36.0). GO analysis was performed by Goatools [92] (github.com/tanghaibao/GOatools). The sequencing data have been deposited at NCBI (Accession number GSE264167), with detailed results available in Supplementary file 1. ATAC-seq. Additionally, RNA-seq analysis was performed by using the same batch of explants to investigate the correlation between chromatin accessibility and gene transcript levels. These data have been deposited at NCBI (Accession number GSE280743), with detailed results available in Supplementary file 1.RNA-seq align with ATAC-Seq.

### Cell and nuclei staining

The cultured calli were sectioned into 5 × 5 × 5 mm pieces and fixed in a 50% FAA solution. Air was removed using a vacuum pump to ensure complete immersion and saturation of samples. The samples were stored at 4°C overnight, then washed through a sequential alcohol series (10, 30, 50, 70, 80, 90, and 100%) for 20 min at each concentration. The dehydrated calli were immersed in a 1:1 solution of Technovit 7100 and anhydrous alcohol overnight at room temperature, and then transferred into pure Technovit 7100 solution containing Hardener I (0.1 g/10 ml) overnight at 4°C. Samples were then embedded and cast with Hardener II (0.1 ml/1.5 ml preparation solution) and incubated in an oven at 42°C for over 2 days. The embedded samples were sectioned and mounted on slides. After complete drying, slides were stained with 0.5% toluidine blue (Sigma-Aldrich, USA) for 30 s and rinsed with distilled water. Callus cells and nuclei were observed under a microscope, and their sizes were measured using an Image J software after photographing. Statistical analysis was conducted using Student’s *t*-test with SPSS (v19.0, 2015, IBM Corp., Armonk, NY).

### *In silico* TFBS analysis

The 2000 bp upstream promoter sequences of DEGs involved in plant regeneration and chromatin organization were retrieved from *N. benthamiana* genome v2.6.1 (Sol Genomics Network). Promoters were scanned for putative cis regulatory elements using PlantCARE [96], which annotates experimentally verified and high confidence predicted motifs without explicit similarity cut offs. In parallel, promoters were analyzed in PlantPAN 4.0 [97]: TFBS detection employed the MATCH algorithm with default thresholds (core similarity = 1.0, matrix similarity = 0.75), and co-occurrence analysis of combinatorial TFBS used a 100-bp distance constraint along with default support and confidence values. Cis element distributions across promoters were plotted using TBtools II for motif mapping and enrichment visualization [98].

### Cas9 *in vitro* cleavage assays

The *in vitro* cleavage assay was performed using the Cas9 *In Vitro* Cleavage Kit according to the manufacturer’s instructions (PC1400, Inovogen Tech. Co., Beijing, China). Briefly, reaction volumes of 22 μl were prepared by mixing 5 μl of positive control SpCas9 protein, 5 μl of positive control gRNA, 10 μl of positive control DNA, and 2 μl of 10× SpCas9 Buffer. The reactions were incubated at 37°C for 10, 20, and 30 min, respectively. Following incubation, the reactions were terminated by heating at 85°C for 10 min, followed by the addition of 5 μl of cleaner and incubation at 55°C for 5 min. Cleavage products (6 μl) were loaded onto a 1.5% agarose gel to assess cleavage efficiency. The intensities of uncut DNA band from the 10-minute cleavage reactions were quantified using ImageJ [47]. Statistical significance was determined by Student’s *t*-test based on four biological replicates.

## Supplementary Material

Web_Material_uhaf240

## Data Availability

The datasets analyzed in this study are publicly available with the accession numbers PRJNA1097301, GSE264167 and GSE280743.
