# Supplementary material for: Auxin affects gene editing efficiency through regulating chromatin accessibility and plant regeneration process
Source: Hortic Res. 2025 Sep 3;12(12):uhaf240. doi: 10.1093/hr/uhaf240 (PMC12685441; doi:10.1093/hr/uhaf240)
Supplement: Web_Material_uhaf240 [file web_material_uhaf240.zip › Supplementary Figures.docx]

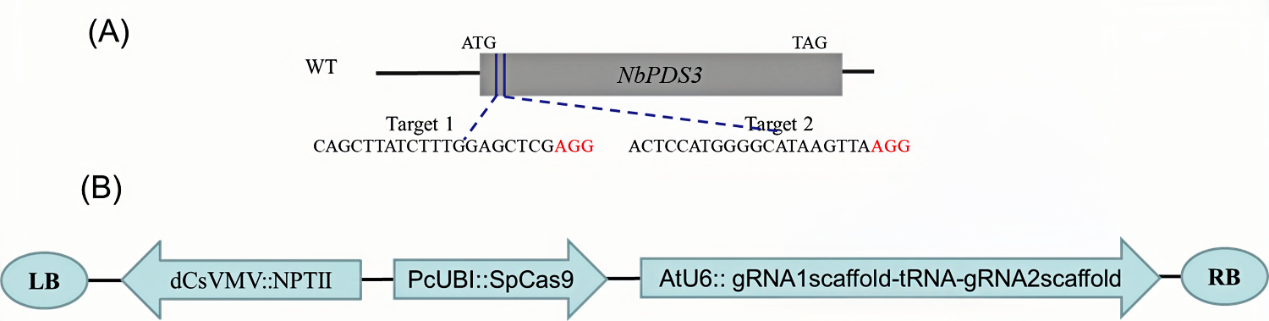
**Supplementary Figures**

**Supplementary Figure S1.** Schematic diagrams for the Cas9/sgRNA vector. (A) The accession number of *PDS* homologous gene in tobacco, and target sites of two sgRNAs. (B) The vector contains an *eGFP::NPT* (*NPTⅡ*: encoding a neomycin phosphotransferase for Kanamycin resistance) fused protein gene driven by a CsVMV promoter. The Cas9 protein is driven by the parsley ubiquitin promoter (PcUBI) and gRNAs were under the control of AtU6 promoter with tRNA cleaving system.


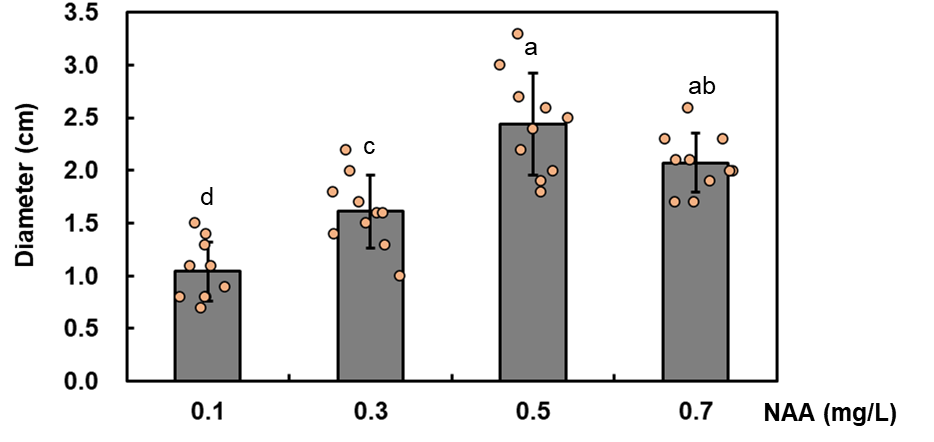


**Supplementary Figure S2.** Diameter of tobacco calli cultured with different NAA concentrations. Ten replicates were included in each group, as shown in Figure 2C. Error bars represent the standard error of the mean. Letters indicate significant difference between groups (ANOVA-LSD, p<0.05).


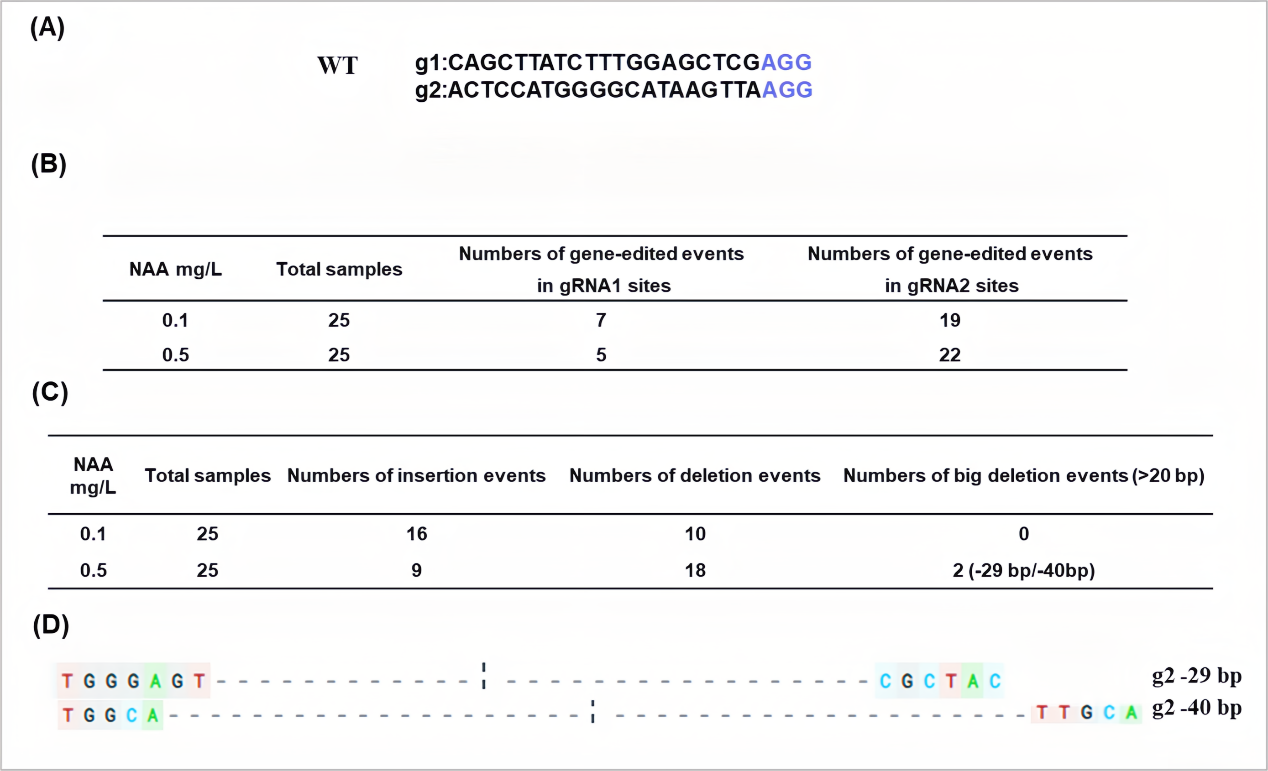
**Supplementary Figure S3.** Mutations at both target sites in *NbPDS3* gene-edited tobacco shoots. (A) Sequence information of wildtype plants at target sites, with PAM sequence highlighted in blue color. (B) The numbers of editing events of *NbPDS3* in the gRNA1 and gRNA2 sites from 25 sequencing samples each from the 0.1 mg/L and 0.5 mg/L NAA treatments. (C) Gene editing results from 25 sequencing samples each from the 0.1 mg/L and 0.5 mg/L NAA treatments. (D) Large deletions detected in 0.5 mg/L NAA treatment group, but not in 0.1mg/L NAA group.


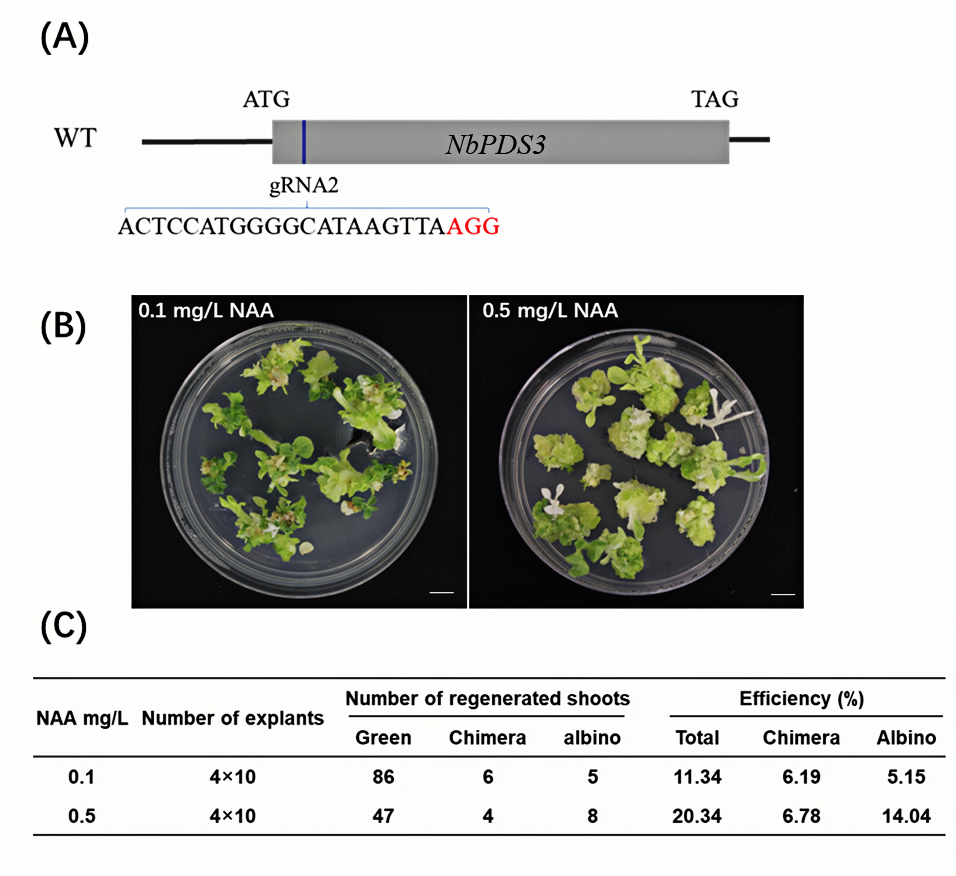


**Supplementary Figure S4.** Effects of auxin concentrations on gene editing efficiency in tobacco with a single gRNA. (A) Schematic diagram for the target *NbPDS3* gene with a single sgRNA. (B) Morphological comparison of tobacco explants treated with 0.1mg/L NAA or 0.5 mg/L NAA, Scale =1 cm. (C) Total number of regenerated shoots and mutant shoots from different NAA concentrations, along with corresponding efficiencies. Four replicates petri dish were applied to each NAA concentration (0.1 and 0.5 mg/L), with each replicate consisting of 10 explants (cotyledons).


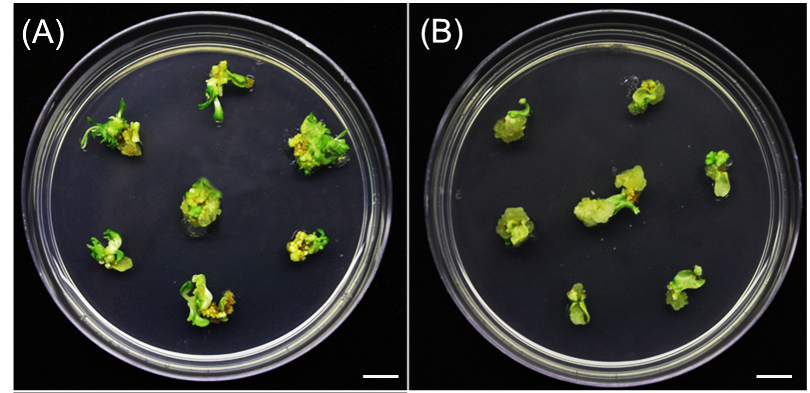


**Supplementary Figure S5**. Morphological comparison of tobacco explants treated with 0.1mg/L NAA (A) and 0.5 mg/L NAA (B) after 25 days of culture, Scale =1 cm.


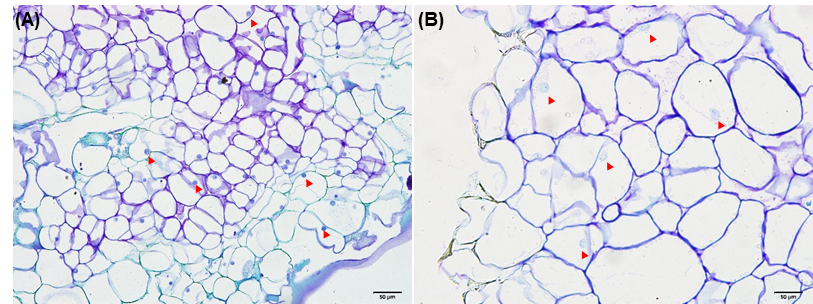


**Supplementary Figure S6.** Histological comparison of tobacco calli cells under 0.1 mg/L (A) and 0.5 mg/L (B) NAA treatment after 25 days of culture. The scale=50μm. Nuclei were indicated by arrowheads.


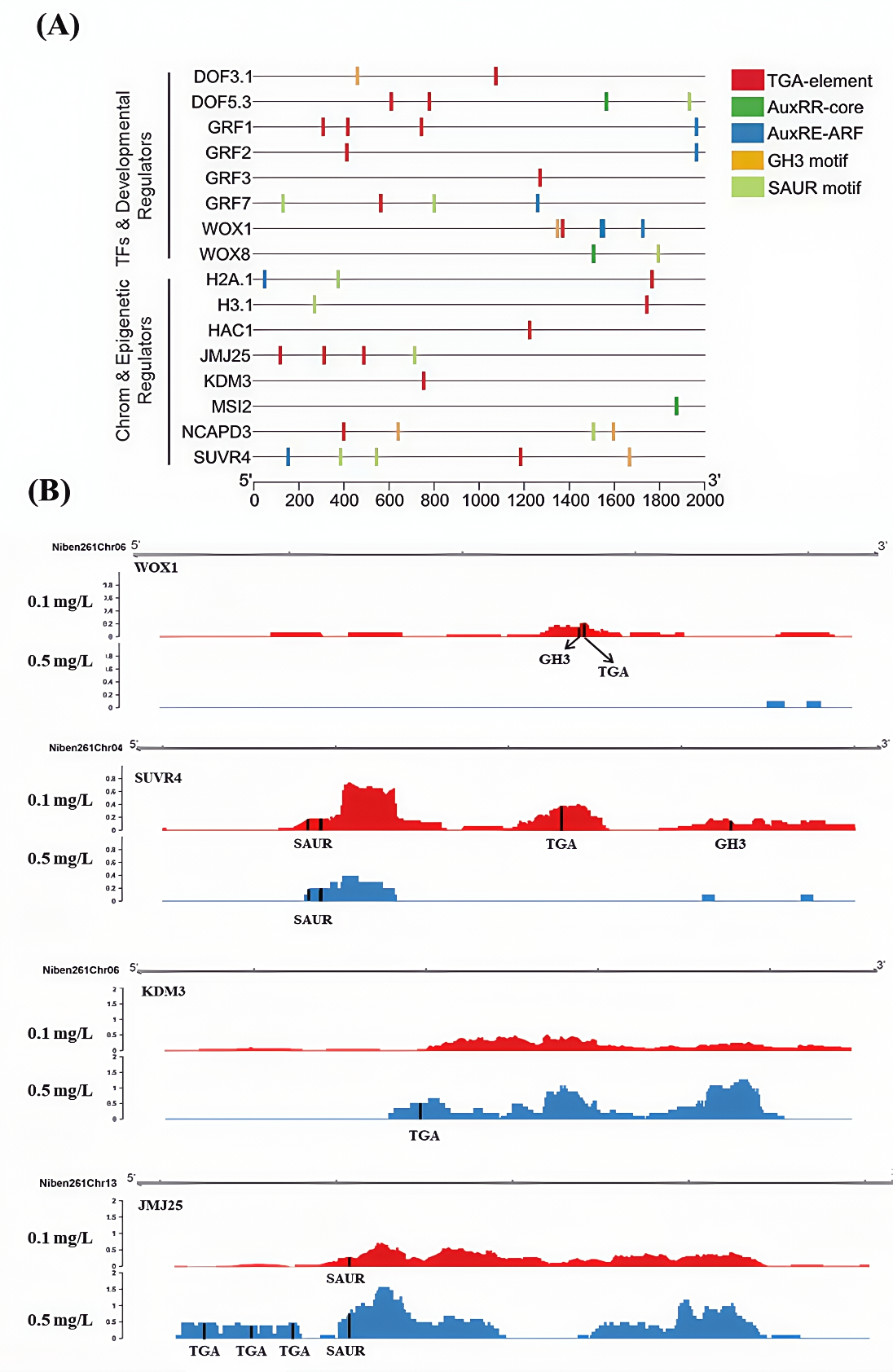


**Supplementary Figure S7.** Integrative analysis of auxin-responsive cis-elements and chromatin accessibility in selected DEGs associated with plant regeneration and chromatin regulation. (A) In silico identification of auxin-responsive transcription factor binding sites in the promoter regions (2 kb upstream of the transcription start site) of differentially expressed genes (DEGs) selected from Supplementary file 1. RNA-seq align with ATAC-seq. Five core auxin-responsive motifs were analyzed: the TGA-element (AACGAC), the AuxRR-core motif (GGTCCAT), the AuxRE-ARF (TGTCTC), the GH3 motif (CATATG) and SAUR motif CACATG). (B) ATAC-seq analysis showing chromatin accessibility patterns in genomic regions surrounding identified auxin-responsive motifs. Data are shown for representative genes including *WOX1*, *SUVR4*, *KDM3*, and *JMJ25*, comparing chromatin openness under low (0.1 mg/L, red) and high (0.5 mg/L, blue) NAA treatment.


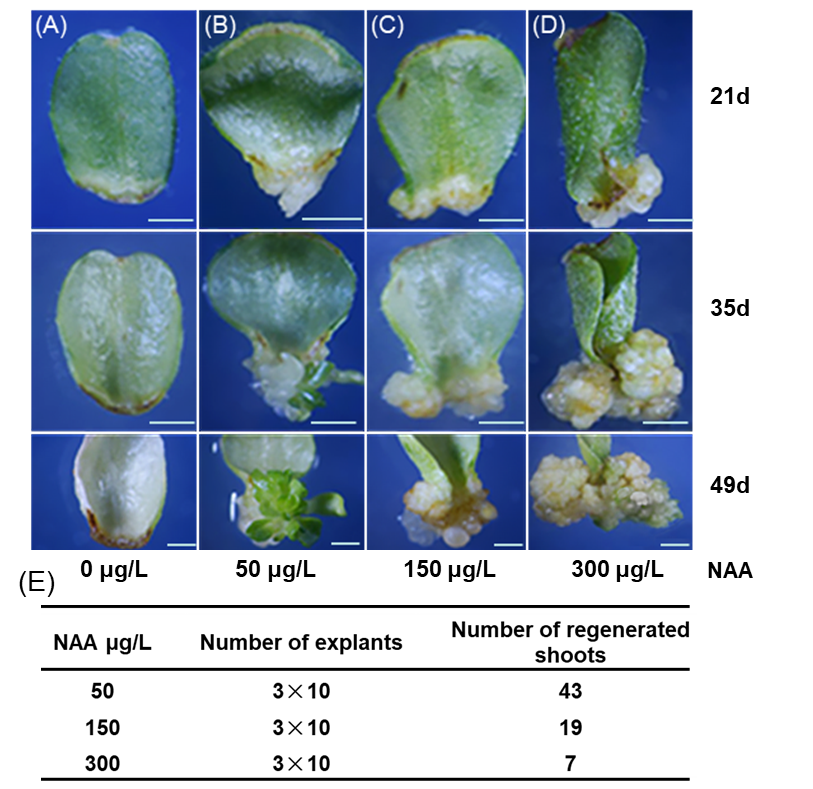


**Supplementary Figure S8.** Effects of different NAA concentrations on tomato callus growth and shoot regeneration. (A) No calli was observed on media without NAA. (B) Rapid shoot initiation occurred at 50 μg/L NAA. (C-D) Tomato calli growth was promoted with the increasing NAA concentrations. (E) Frequence of shoot regeneration under different NAA treatments. Explants were continuously cultured on media containing 2 mg/L ZT and different concentrations of NAA. The scale=5mm.


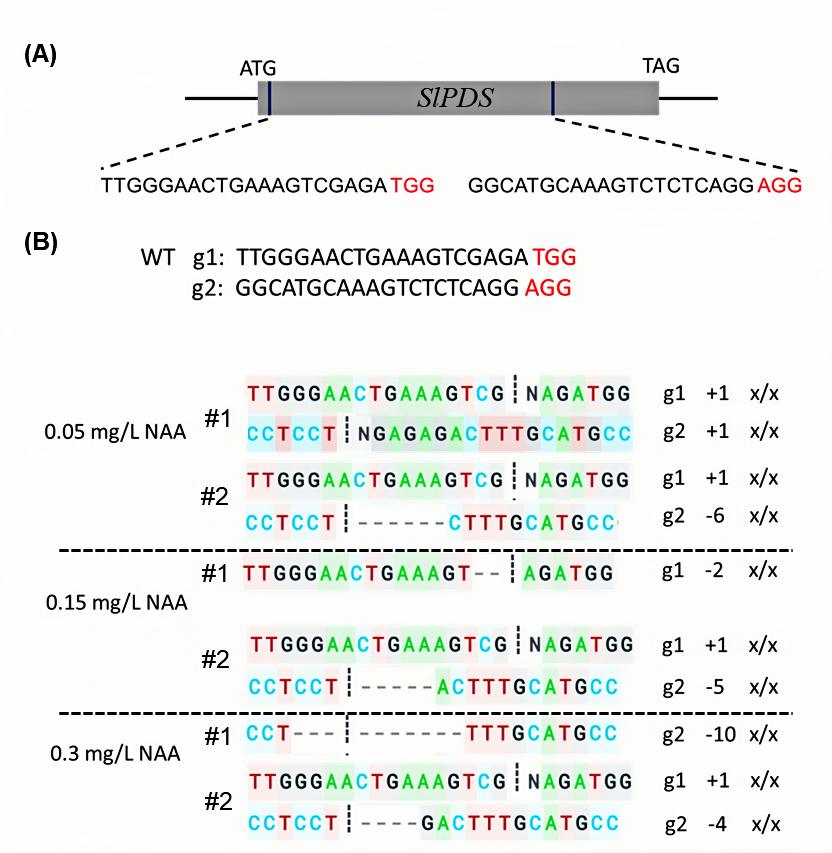


**Supplementary Figure S9.** Mutations at both target sites in gene-edited tomato shoots. (A) The accession number of *PDS3* homologous gene in tomatoes and target sites of two sgRNAs. (B) Sequences of two sgRNAs, with PAMs highlighted in red, and editing patterns of *PDS3* in the mutant shoots. “-” and “” denote nucleotide deletions and insertions, respectively. Two representative mutant samples from each NAA concentration were randomly selected for PCR sequencing, x/x represents that both alleles were edited at the target site in tomato.


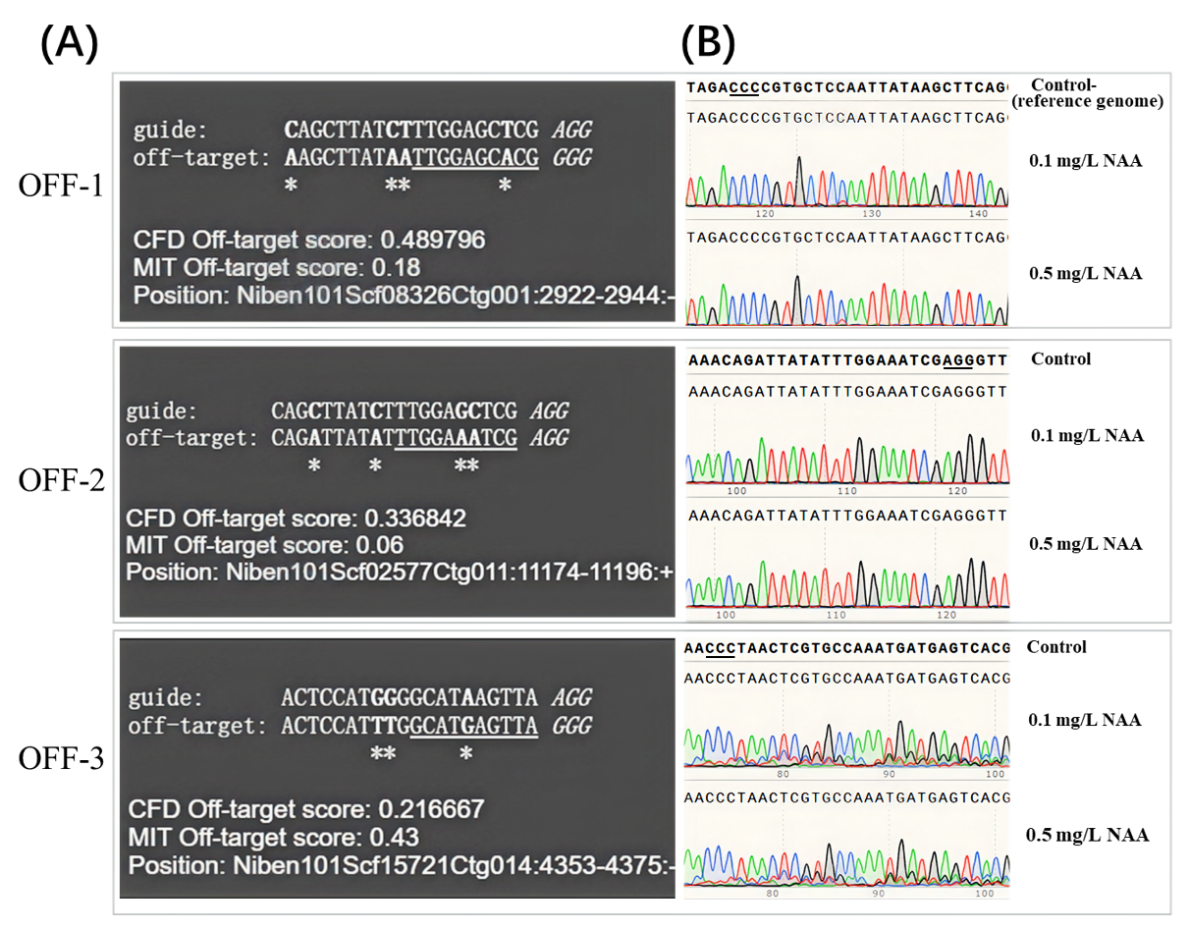


**Supplementary Figure S10.** Sanger sequencing of potential off-target activities in gene-edited tobacco. (A) Predicted off-target positions by CRISPOR. (B) No off-target events were detected in both 0.1mg/L NAA and 0.5mg/L NAA treatment groups. The reference genome sequences were used as controls, the PAMs (NGG) were indicated by black underlines in B.


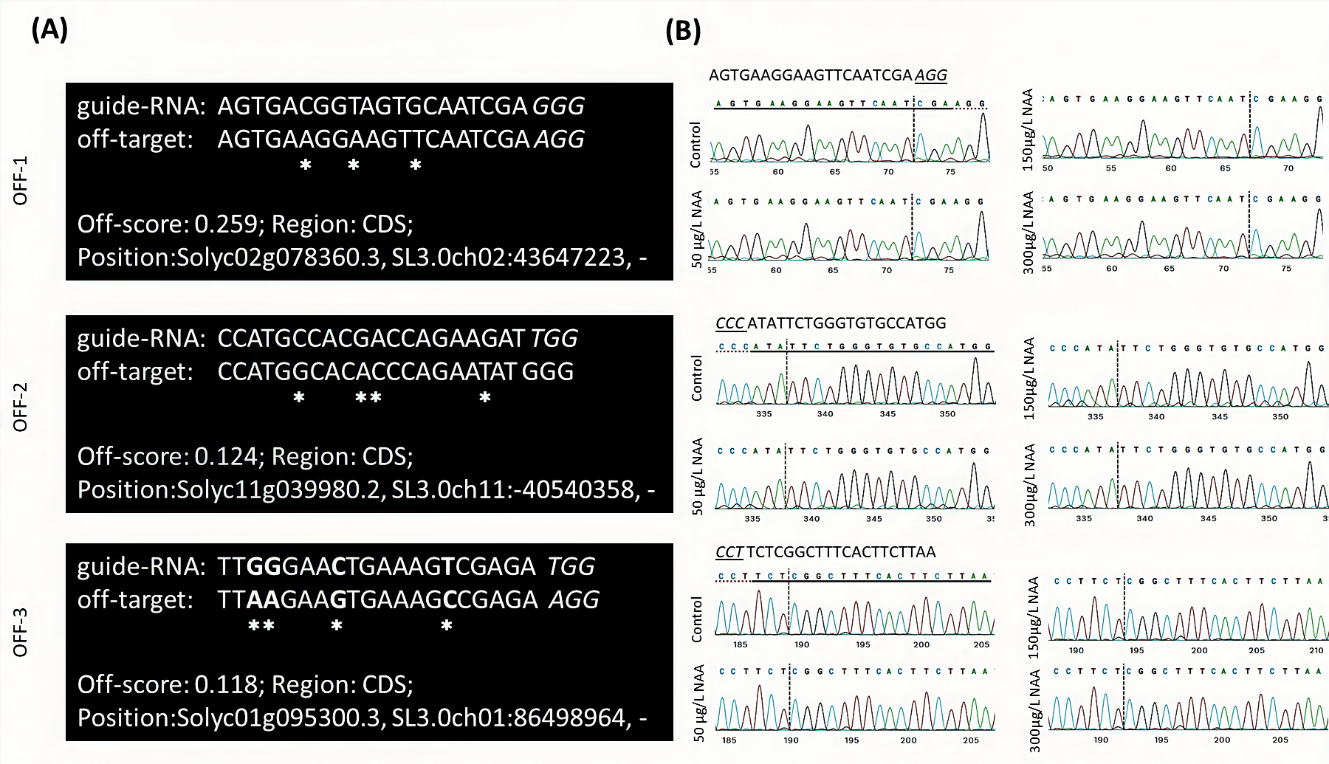


**Supplementary Figure S11.** Sanger sequencing of potential off-target activities in gene-edited tomato. (A) Predicted off-target positions identified using CRISPOR. (B) No off-target events were detected in 50 μg/L NAA, 150μg/L NAA and 300μg/L NAA treatments. Sequencing results of a wild type tomato plant were used as controls. The PAMs (NGG) were indicated by black underlines in B.


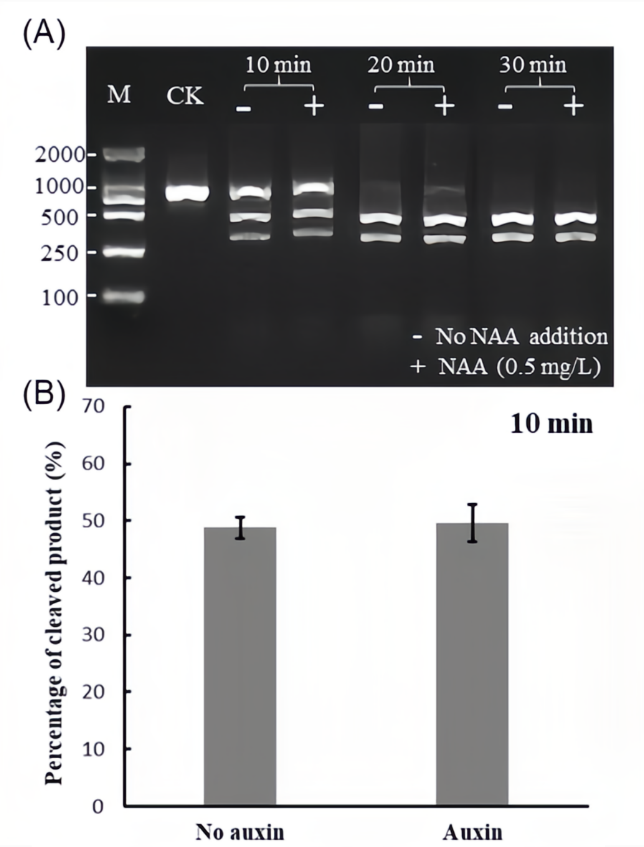


**Supplementary Figure S12**. Auxin does not directly affect Cas9 enzymatic activity *in vitro*. (A) *In vitro* cleavage assay by incubating the positive control DNA containing a single target site, corresponding sgRNA and Cas9 protein. Cleavage of the substrate DNA yields fragments of 310 bp and 450 bp. These reactions were conducted with or without NAA (0.5 mg/L) and were terminated after 10, 20 and 30 minutes, followed by analysis on 1.5% agarose gels. CK: linearized target DNA without Cas9 treatment (uncut control). (B) Quantification of Cas9 cleavage efficiency after 10 minutes of reaction. Data represent mean values ± standard error from four replicates, no significant difference was observed between reactions with and without 0.5 mg/L NAA addition (Student’s *t*-test, p>0.05).
