# Supplementary material for: Auxin affects gene editing efficiency through regulating chromatin accessibility and plant regeneration process
Source: Hortic Res. 2025 Sep 3;12(12):uhaf240. doi: 10.1093/hr/uhaf240 (PMC12685441; doi:10.1093/hr/uhaf240)
Supplement: Web_Material_uhaf240 [file web_material_uhaf240.zip › Supplementary Tables.docx]

**Supplementary Table S1**. The differentially expressed genes and corresponding gene loci in tobacco reference genome.

| The name of gene | Gene locus | The name of gene | Gene locus |
| --- | --- | --- | --- |
| *H2A.1* | Niben261Chr04g1569006 | *MCC1* | Niben261Chr19g0797004 |
| *H2X.like* | Niben261Chr10g0079012 | *WDR5* | Niben261Scf16471g0000003 |
| *H3.1* | Niben261Chr05g1100011 | *ELF6* | Niben261Chr10g0902010 |
| *H4* | Niben261Chr14g0664003 | *REF6* | Niben261Chr06g0615010 |
| *HAT2* | Niben261Chr11g0012005 | *KDM3* | Niben261Chr06g0757005 |
| *HMGYA_1* | Niben261Chr19g1378006 | *HAC1* | Niben261Chr18g0699016 |
| *NCAPD3* | Niben261Chr17g0034007 | *LDL3* | Niben261Chr11g0626003 |
| *WUS* | Niben261Chr16g0019003 |  |  |
| *WOX1* | Niben261Chr06g1145001 |  |  |
| *GRF1* | Niben261Chr15g0927001 |  |  |
| *GRF2* | Niben261Chr16g0073012 |  |  |
| *GRF3* | Niben261Chr13g0240003 |  |  |
| *GRF4* | Niben261Chr18g1098001 |  |  |
| *GRF5* | Niben261Chr05g1295014 |  |  |
| *GRF6* | Niben261Chr19g0457006 |  |  |
| *GRF7* | Niben261Chr08g0397017 |  |  |
| *GRF8* | Niben261Chr14g1535009 |  |  |
| *GRF9* | Niben261Chr09g1183014 |  |  |
| *GIF* | Niben261Chr16g0484013 |  |  |
| *BARD1* | Niben261Chr15g1495004 |  |  |
| *SUVR4* | Niben261Chr04g1116002 |  |  |
| *DNMT1* | Niben261Chr02g0106024 |  |  |
| *WOX3-LIKE* | Niben261Chr03g1155012 |  |  |
| *MSI2* | Niben261Chr09g1251007 |  |  |
| *WOX4* | Niben261Chr11g0408004 |  |  |
| *WOX8* | Niben261Chr15g1054006 |  |  |
| *WOX9* | Niben261Chr07g1299001 |  |  |
| *WOX11* | Niben261Chr08g1050007 |  |  |
| *PLT3* | Niben261Chr07g0790007 |  |  |
| *PLT6* | Niben261Chr15g1229010 |  |  |
| *DOF3.1* | Niben261Chr15g1136003 |  |  |
| *DOF5.3* | Niben261Chr01g0742001 |  |  |
| *HDA2* | Niben261Chr03g1528011 |  |  |
| *JMJ30* | Niben261Chr02g0077015 |  |  |
| *JMJ16* | Niben261Chr11g1348015 |  |  |
| *JMJ25* | Niben261Chr13g1182002 |  |  |
| *ADA2* | Niben261Chr11g0355004 |  |  |

**Supplementary Table S2.** List of PCR primers used for detecting gene-edited events by Sanger sequencing in tobacco and tomato.

| **Name** | **Strand** | **Sequence** |
| --- | --- | --- |
| *NbPDS3*_1 | F | 5’- CTTGATTTTGTGGGTGAAGGA -3’ |
|  | R | 5’- ATGGTTTAGTTGGGCGTGAG-3’ |
| *NbPDS3*_2 | F | 5’- ACAACTCCTAGGCGGTTTCA-3’ |
|  | R | 5’- GAATGATCTTCCCTCCGAAA-3’ |
| *SlPDS*_1 | F | 5’- CATGCAAATGGGCCTCTTGT-3’ |
|  | R | 5’-GGTACTCCGACTAACTTCTCCA-3’ |
| *SlPDS*_2 | F | 5’- GGTGCTTTCATTCGTTCCGA-3’ |
|  | R | 5’-GACCCGGAATATCACCTGCA-3’ |

**Supplementary Table S3**. List of PCR primers used for detecting off-target effects by Sanger sequencing in tobacco.

| **Name** | **Strand** | **Sequence** |
| --- | --- | --- |
| NbOFF-1 | F | 5’- GTTCGCTGTGATATATTAGC -3’ |
|  | R | 5’- CCAGAGGATGCATCACTGCA-3’ |
| NbOFF-2 | F | 5’- TGAGTTAGTTGTGCATCGCG -3’ |
|  | R | 5’- CTTATCCCAATTGCTCTAC-3’ |
| NbOFF-3 | F | 5’- CCTAACCCAACTAATCTAAC-3’ |
|  | R | 5’- GTATAGGCCATCTATGGCCT-3’ |

**Supplementary Table S4.** List of PCR primers used for detecting of off-target effects by Sanger sequencing in tomato.

| **Name** | **Strand** | **Sequence** |
| --- | --- | --- |
| SlOFF-1 | F | 5’- TCGTATTGTTCTTTGAGGGTATTTAC-3’ |
|  | R | 5’- CCCAACAAGAACATATCAAGCA-3’ |
| SlOFF-2 | F | 5’- CCCAACAAGAACATATCAAGCA -3’ |
|  | R | 5’- TGGCCAATACCCCTTCAACT-3’ |
| SlOFF-3 | F | 5’- CCTGATTGGTGGAAGTTGGC-3’ |
|  | R | 5’- TATCCAGACGACAACCCCAC-3’ |
